# Recurrently connected and localized neuronal communities initiate coordinated spontaneous activity in neuronal networks

**DOI:** 10.1371/journal.pcbi.1005672

**Published:** 2017-07-27

**Authors:** Davide Lonardoni, Hayder Amin, Stefano Di Marco, Alessandro Maccione, Luca Berdondini, Thierry Nieus

**Affiliations:** Neuroscience and Brain Technologies Dept., Fondazione Istituto Italiano di Tecnologia, Genoa, Italy; Radboud Universiteit Nijmegen, NETHERLANDS

## Abstract

Developing neuronal systems intrinsically generate coordinated spontaneous activity that propagates by involving a large number of synchronously firing neurons. *In vivo*, waves of spikes transiently characterize the activity of developing brain circuits and are fundamental for activity-dependent circuit formation. *In vitro*, coordinated spontaneous spiking activity, or network bursts (NBs), interleaved within periods of asynchronous spikes emerge during the development of 2D and 3D neuronal cultures. Several studies have investigated this type of activity and its dynamics, but how a neuronal system generates these coordinated events remains unclear. Here, we investigate at a cellular level the generation of network bursts in spontaneously active neuronal cultures by exploiting high-resolution multielectrode array recordings and computational network modelling. Our analysis reveals that NBs are generated in specialized regions of the network (functional neuronal communities) that feature neuronal links with high cross-correlation peak values, sub-millisecond lags and that share very similar structural connectivity motifs providing recurrent interactions. We show that the particular properties of these local structures enable locally amplifying spontaneous asynchronous spikes and that this mechanism can lead to the initiation of NBs. Through the analysis of simulated and experimental data, we also show that AMPA currents drive the coordinated activity, while NMDA and GABA currents are only involved in shaping the dynamics of NBs. Overall, our results suggest that the presence of functional neuronal communities with recurrent local connections allows a neuronal system to generate spontaneous coordinated spiking activity events. As suggested by the rules used for implementing our computational model, such functional communities might naturally emerge during network development by following simple constraints on distance-based connectivity.

## Author summary

Coordinated spontaneous spiking activity is fundamental for the normal formation of brain circuits during development. However, how ensembles of neurons generate these events remains unclear. To address this question, in the present study, we investigated the network properties that might be required to a neuronal system for the generation of these spontaneous waves of spikes. We performed our study on spontaneously active neuronal cell cultures using high-resolution electrical recordings and a computational network model developed to reproduce our experimental data both quantitatively and qualitatively. Through the analysis of both experimental and simulated data, we found that network bursts are initiated in regions of the network, or “functional communities”, characterized by particular local connectivity properties. We also found that these regions can amplify the background asynchronous spiking activity preceding a network burst and, in this way, can give rise to coordinated spiking events.

As a whole, our results suggest the presence of functional communities of neurons in a developing neuronal system that might naturally emerge by following simple constraints on distance-based connectivity. These regions are most likely required for the generation of the spontaneous coordinated activity that can drive activity-dependent circuit formation.

## Introduction

Neuronal systems, including brain circuits’ *in vivo* and cultured networks, intrinsically generate coordinated and spatiotemporally propagating spontaneous spiking activity during their development [1]. This type of spontaneous activity (or “waves”) has been studied in several brain circuits, including the cerebellum [2], the hippocampus [3,4], the thalamus [5] and in sensory systems such as the retina [6]. These studies have shown that coordinated spontaneous waves of spikes transiently characterize the firing regime of early developing brain circuits and are associated with activity-dependent circuit formation [7,8]. For instance, in the visual sensory system, waves of spikes are required for the normal development of sensory representations before visual experience formation, and several studies have investigated the underlying cellular and molecular mechanisms that can change the dynamics of these spontaneous waves [6,9]. Indeed, as brain circuits form, the spatiotemporal dynamics of the coordinated spiking activity changes and characterizes different stages of development. Successively, through the effect of neuromodulation, the spiking activity switches from a coordinated firing regime to a regime characterized by asynchronous spiking neurons [1,7].

Interestingly, neurons *in vitro* can also self-organize and rewire to form networks [10,11] that spontaneously express a rich repertoire of coordinated spiking activity after a few weeks of growth. The network activity of these isolated neuronal systems does not switch to a sparse spiking regime as occurs *in vivo*, and collective spiking patterns can rather persist over time, lasting up to a few months [12,13]. During collective spiking events, referred to as network bursts (NBs) [14], most neurons in the network fire together [15,16]. As clearly unveiled only recently with high-resolution electrical recordings [17,18] or Ca^2+^ functional imaging [19], cultured networks also propagate waves of spikes.

Even though the specific dynamic of these spiking waves varies depending on the neuronal system and point in development, all of these spontaneous coordinated events share remarkably similar macroscopic properties. Indeed, these events typically last up to a few hundreds of milliseconds, spatiotemporally propagate through the network and occur with an interval in the order of minutes. Additionally, the number of spatiotemporal patterns tends to be restricted to a few classes, each one having distinct regions that initiate these propagating events. However, despite the large interest in the dynamics of this type of activity, the mechanism by which a neuronal system can generate coordinated spiking events and whether the regions initiating these events require specific cellular or connectivity properties remain unclear. Previous works have suggested that the presence of hub neurons [20] and the local cellular properties of sub-populations of neurons [21] might underlie the sub-networks that act as nucleation centres of coordinated spiking activity events.

Here, we investigate the generation of NBs in primary neuronal cultures by combining high-resolution electrical recordings and computational modelling. Neurons in culture form isolated networks that can intrinsically generate coordinated spiking activity interleaved with phases of asynchronous spikes and offer several advantages for our study. Indeed, while coordinated spiking activity in developing brain circuits has been suggested to depend on the specific topological properties of the circuit (hub neurons in the hippocampus or short connections between Purkinje cells in the cerebellum) [20], cultured networks can display this type of spontaneous activity without a clear physiological organization of the cellular network topology. Additionally, neuronal cultures are the neuronal system that currently offers the lowest undersampling of firing neurons when recorded with high-resolution multielectrode arrays (MEAs) [22] that consist of complementary metal-oxide-semiconductor (CMOS) devices [17,23]. The detailed and simultaneous access to the spiking activity of several thousands of neurons provided by 4096-electrode CMOS-MEAs allows for the fine quantification of mean activity parameters [22,24], the tracking of spatiotemporal propagations and the localization of the sites generating the NB events [18].

To evaluate the potential contribution to the generation of NBs of different and experimentally hidden structural and functional variables, we combined the analysis of high-resolution electrical recordings with similar analyses of simulated data. The computational network model developed in this work consists of 4096 conductance-based point-process neurons [25] connecting near neurons with a higher probability than far neurons. This rule was found to enable our model to both quantitatively and qualitatively mimic the experimental spontaneous spiking activity of cultured neuronal networks. The model was also further assessed by comparing simulated and experimental data under conditions of pharmacological manipulation of synaptic transmission, thus also allowing for the verification of the role of α-amino-3-hydroxy-5-methyl-4-isoxazolepropionic acid (AMPA), γ-aminobutyric acid (GABA) and N-methyl-D-aspartic acid (NMDA) currents in the generation of NBs and in shaping their dynamics. We then used the experimental data and the full details of our model to investigate the connectivity properties of the network regions associated with the generation of NBs (or ignition sites, ISs). This allowed us to identify functional communities (fCOMs) of neurons associated with the regions initiating NBs, to characterize their structural and functional properties and to suggest a possible mechanism for the local initiation of NBs that relies on the connectivity properties of the fCOMs. Finally, as previously suggested in [21], by analysing the temporal motifs of the spike patterns, we investigated whether the spiking activity preceding an NB can be predictive of the following NB.

## Results

### High-resolution recordings of spontaneous activity in neuronal cultures

**Fig 1:**
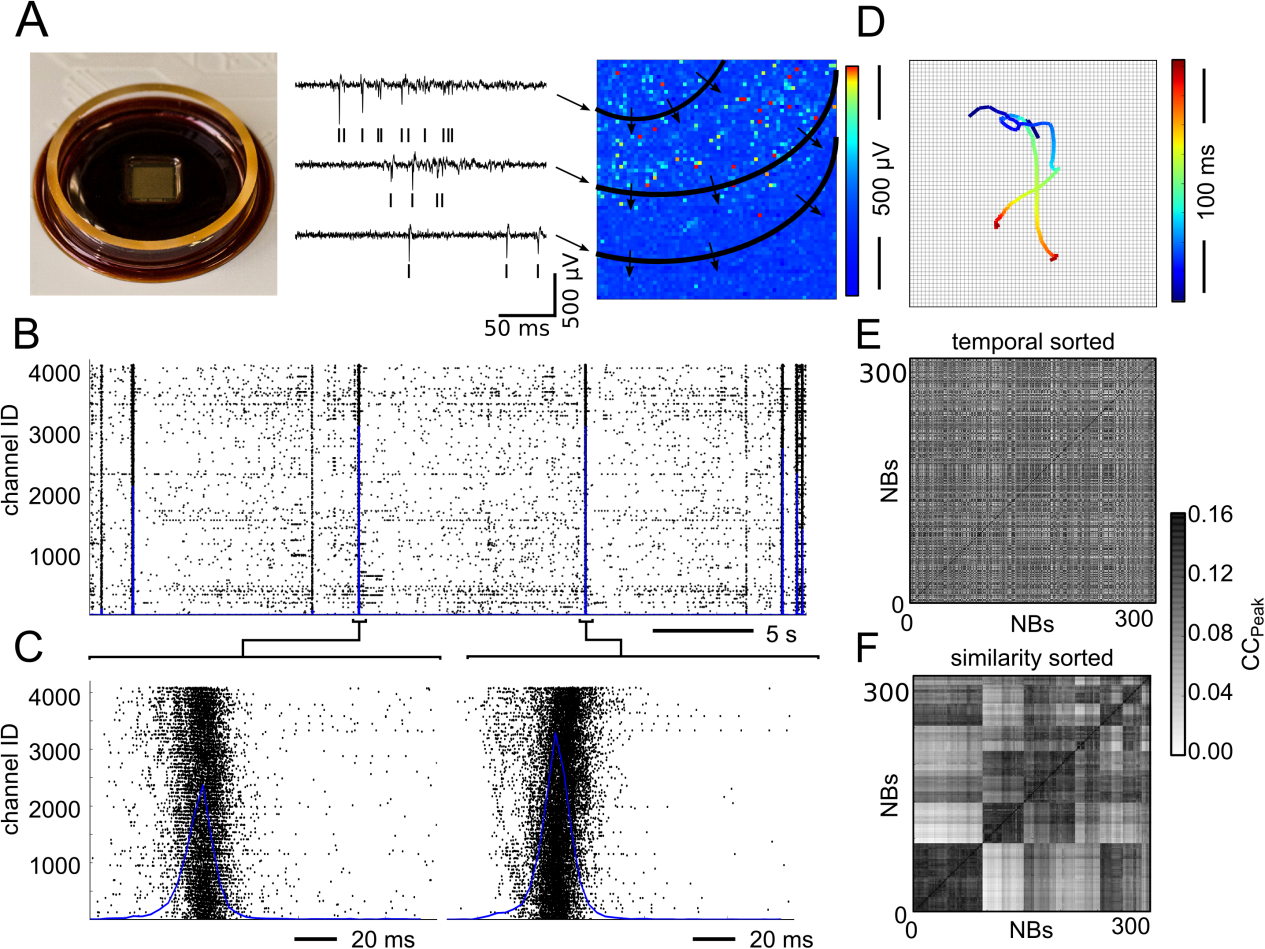
Spontaneous spiking activity recorded with CMOS-MEAs in hippocampal cultures. (A) High-density CMOS-MEAs simultaneously record extracellular electrical signals from an array of 64 x 64 electrodes covering a 5.12mm x 5.12mm area (three representative traces in black). Coordinated spontaneous spiking activity, or network bursts (NBs), propagates through the network, as indicated by the temporal differences in the spiking activity (black ticks). The three traces are part of the propagating activity shown on the right. (B) Raster plot of 40 s of spontaneous activity. The spike count (blue line, 5-ms time bins) displays a peak in correspondence of the NBs. (C) Raster plots and (D) Centre-of-activity trajectories (CATs, time of the propagation is colour coded) of two consecutive NBs exhibiting different propagations lasting approximately 100 ms. (E) The cross-correlation matrix of NBs shows that events of the same cluster do not occur with a periodicity. (F) Instead, the reordered cross-correlation matrix of NBs shows that the NBs are clustered in a few classes.

The spontaneous electrophysiological activity of 15 hippocampal neuronal cultures (19–21 days *in vitro*, DIVs) was recorded at high resolution with the 4096 electrodes of CMOS-MEAs (Fig 1A). The recordings show two alternating firing regimes consisting of long epochs of sparse asynchronous spiking lasting several seconds interleaved with short periods of NB events, see Fig 1B. During an NB, a large fraction of neurons in the network fired action potentials at a high rate (≃ 100 Hz) for a short time (<100 ms). In contrast, before and after an NB, neurons rarely fired action potentials and showed a low mean firing rate (MFR) (≃ 0.1*Hz*). As shown in Fig 1C, NBs comprised the sequential activation of neighbouring neurons recruited to propagate the spiking activity throughout the entire network. The origin and the propagation trajectory of each NB was estimated by computing the average position of the spiking activity over time using a centre-of-activity trajectory (CAT) analysis [18]. The results of this analysis are reported in Fig 1D for the two NBs shown in Fig 1C. Based on the CAT analysis [26], NBs could be sorted into a few classes (i.e., < 10) of spatiotemporal patterns, with each class including events sharing a similar average propagation trajectory and origin. In accordance with previously reported data [15], the cross-correlation matrix of NBs (Fig 1E and 1F) computed on our high-resolution recordings shows that NB classes are preserved along the whole recording duration and that the temporal sequence of these different NBs does not show any recognizable time-periodicity or class-related structure [15,18].

### Simulations of spontaneous and pharmacologically manipulated neuronal culture activities

We developed a computational network model to investigate experimentally hidden network variables based on the recorded experimental activity. Briefly, this network model (see the Materials and Methods section for more details) consisted of 4096 conductance-based point-process neurons [25] with a connectivity determined using a Gaussian radial basis function. Other connectivity rules, such as the random and radius graphs, were discarded because they were unable to reproduce the propagation patterns and velocities of the experimental NBs (see S1 Appendix for a comparison with other network topologies). In the model, each neuron receives independent Poisson inputs that generate fluctuations in the membrane potential and lead to asynchronous spiking activity in the network.

**Fig 2:**
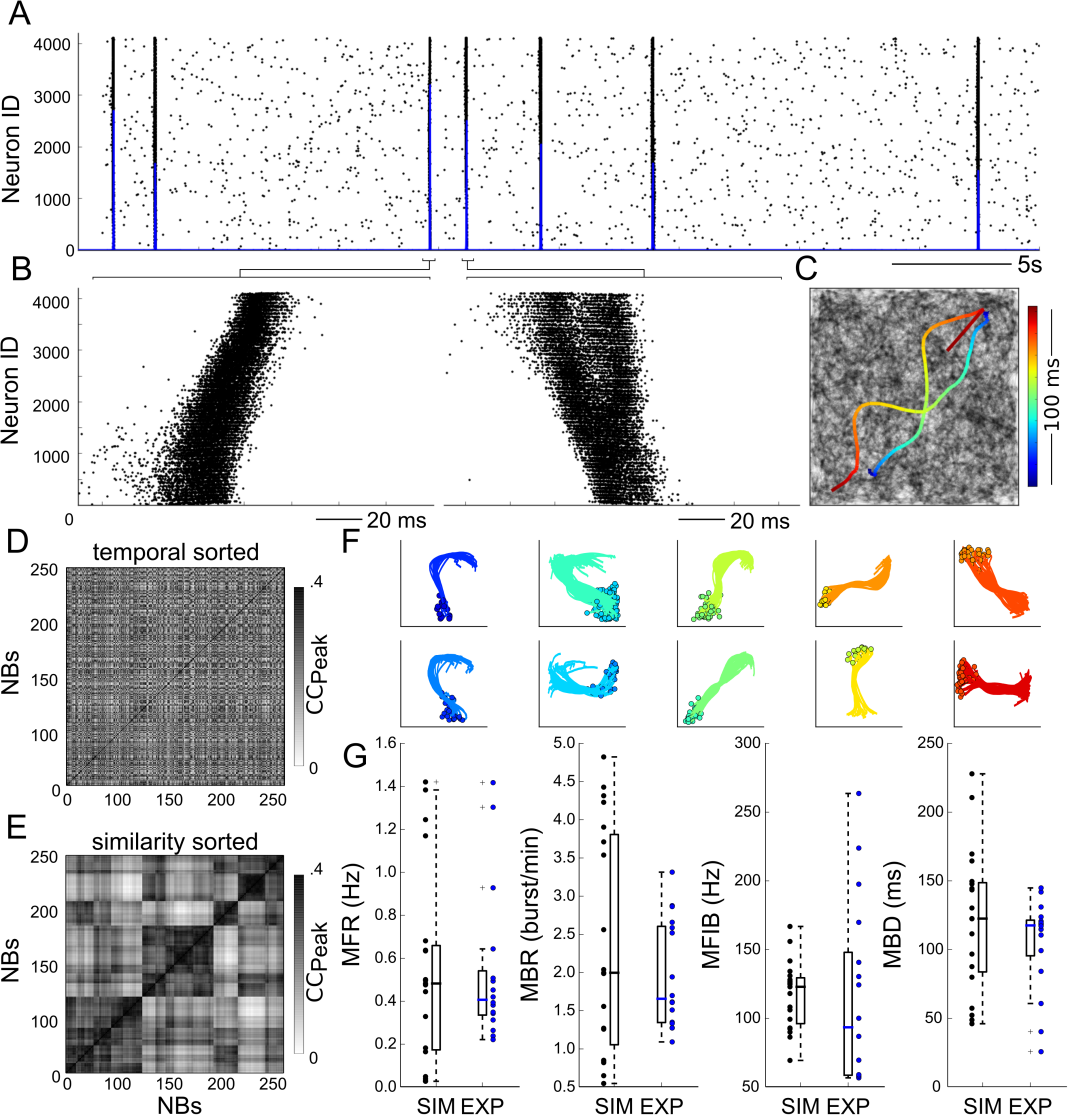
Simulated spontaneous spiking activity in the neuronal network model. (A) Raster plot of spiking activity displaying networks bursts (NBs) comparable to the experimental ones (c.f. Fig 1A). (B) Close-up on two subsequent NBs showing clear spatiotemporal propagations. (C) Plots of the CATs for the two NBs shown in B. (D) Cross-correlation matrix of NBs showing the absence of any temporal correlation among NBs, as in the experimental data (c.f. Fig 1E). (E) The reordered cross-correlation matrix of simulated NBs shows similar features to the one computed for the experimental data (c.f. Fig 1F). (F) Clustered CATs share common ignition sites (circles) and propagating paths through the network. (G) The activity parameters, mean firing rate (MFR), mean bursting rate (MBR), mean firing intra-burst (MFIB) and mean burst duration (MBD) are in accordance with the experimental data (n = 15 experimental recordings, n = 20 simulations, p-values 0.27, 0.97, 0.95, and 0.35, respectively).

The simulated spontaneous network activity was qualitatively comparable to the activity of our experimental recordings (compare Fig 2A with Fig 1B). In addition, the adopted connectivity rule enabled the generation and propagation of NBs throughout the entire network (Fig 2B and 2C) and mimicked our experimental recordings (Fig 1B and 1C). As shown in the raster plots, different neurons involved in an NB fired with different timings depending on their recruitment in the NB propagation. As observed in the experimental data, the simulated NBs also did not show any recurrent or periodic organization (Fig 2D). Simulated NBs could be clustered in a few classes of spatiotemporal patterns (Fig 2E and 2F) based on the CAT analysis as in the previous experiments and in [18]. Finally, the statistical results of the simulated network activities were in accordance with those obtained from the experimental data (Fig 2G), including the MFR, the mean bursting rate (MBR), mean firing intra-burst (MFIB) and mean burst duration (MBD) (see the Materials and Methods section). These results show that our computational network model can spontaneously generate NBs as expressed by neuronal cultures in our recordings and similar to NBs reported in previous studies [12,27,28].

**Fig 3:**
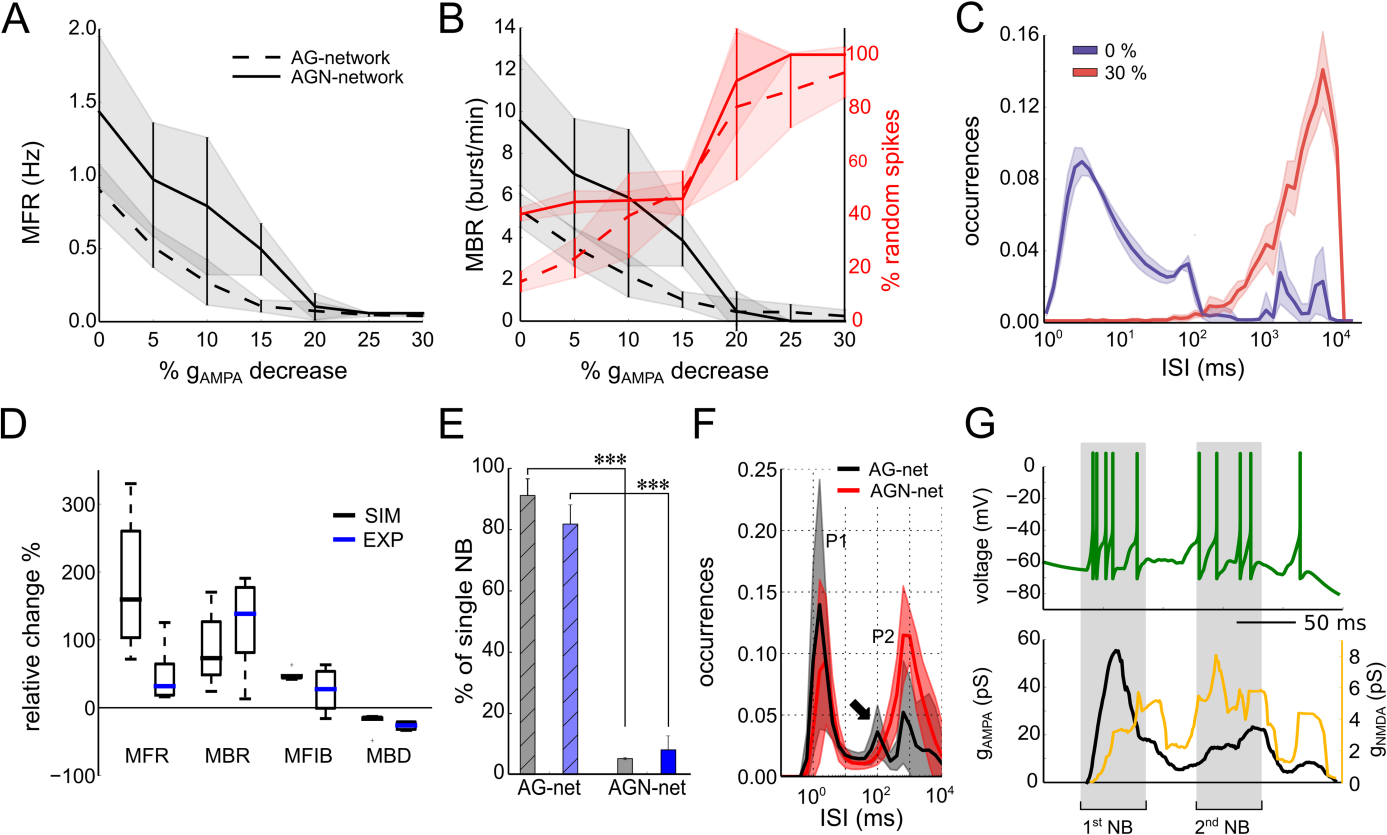
Effects of synaptic transmission on network bursts. (A) Mean firing rate (MFR) as a function of the decreasing AMPA conductance in AG-networks (i.e., networks with AMPA and GABA conductance, dashed lines) and AGN-networks (i.e., networks with AMPA, GABA and NMDA conductance, solid lines). (B) The reduction in AMPA conductance decreases the mean bursting rate (MBR) and increases the number of asynchronous spikes (random spikes). (C) The reduction in AMPA conductance determines a shift in the ISI distribution, from a multi-peak (0% AMPA reduction) to a single-peak distribution (30% AMPA reduction). (D) The model predicts changes in the activity parameters (MFR, MBR, MFIB, MBD), reproducing recordings under pharmacological blockade of inhibition with bicuculline (BIC, 30 μM, p-values: 0.052, 0.473, 0.189, and 0.449, respectively; independent t-tests, n = 5 simulations, n = 4 recordings). (E) AG-networks show single NBs, while AGN-networks show superbursts, both in recordings (n = 3, gray) and in simulations (n = 10, blue). (F) In AGN-networks, (black, n = 10), the ISI distribution has three peaks: P1 relates to the firing within an NB, P2 to the firing between consecutive NBs, and the peak highlighted by a solid arrow (at approximately 100 ms) to the time intervals across consecutive NBs of a superburst. Blockade of NMDA (AG-network) removes the latter peak from the distribution (red, n = 10). (G) Exemplary simulated trace of the membrane potential of a single cell during a superburst and total conductance of AMPA (black) and NMDA (orange) for the same neuron. Note that during the first NB, the NMDA conductance is negligible compared with that of AMPA.

To further validate our model, we assessed its performance in reproducing pharmacologically manipulated network activity. Given that synaptic signalling is a fundamental component in the relay of information among neurons in a network, we investigated its role in the dynamics of NBs by exploring the contribution of three main excitatory and inhibitory currents (i.e., AMPA, NMDA and GABA). In accordance with previous works [29,30], we observed that our computational network model endowed with AMPA and GABA conductance (or AG-networks) could trigger NBs. Experimentally, impairing excitatory synaptic transmission with CNQX (a selective blocker of AMPA receptors) caused the silencing of NBs [31,32,33]. To understand the extent to which manipulations of AMPA can affect NBs, we tested the response of the AG-network by decreasing AMPA conductance from 48 to 30 *μS*, leaving GABA inhibition unaltered. Our simulation results show that a decrease in AMPA induced a reduction in spontaneous spiking activity, as quantified in Fig 3A with the MFR. For low values of AMPA conductance (< 38 *μS*), the network stopped firing NBs (MBR was close to zero; Fig 3B, black dashed lines), and the percentage of random spikes (not belonging to a burst) were predominant (Fig 3B, red dashed lines). Under this low-AMPA condition, the addition of NMDA currents (or AGN-network) partially compensated for the reduction in AMPA excitation (Fig 3A and 3B, solid lines). Indeed, we observed that for a 30% decrease in AMPA conductance, the spiking activity of the AGN-networks became very sparse, with inter spike intervals (ISIs) of several seconds (Fig 3C, red distribution). Conversely, in AGN-networks in the control condition (0% decrease in AMPA conductance), the ISI distribution (Fig 3C, blue line) showed both short (< ~100 ms) and long (> ~500 ms) ISIs, indicating the presence of NBs interleaved with asynchronous spikes. Notably, previous experimental observations [34] reported that homeostatic changes in AMPA currents comparable to those of our results are required to drive the network from uncoordinated to coordinated firing regimes.

In addition to the effects of AMPA, we investigated those of GABA inhibition on NBs, both experimentally by blocking GABA-receptors with bicuculline (n = 4 recordings, 30 μM) and with simulations (n = 5) by setting a null GABA conductance in the model. The blockade of GABA currents has previously been shown to not prevent the firing of NBs in cell culture networks [35,36,37]. Compared with the results in control conditions (i.e., unblocked GABA; “GABA-ON”), the blockade of inhibitory synapses in the model (GABA-OFF) caused an increase in the MFR, MBR, MFIB and a decrease in the MBD, both in our recordings and in simulations (Fig 3D; for further details see S5 Appendix).

We finally investigated the impact of NMDA on the dynamics of the network activity. Experimentally, the main consequence of blocking NMDA currents (50 μM of APV, n = 3) was the suppression of their superburst firing regime [12](sequences of NBs interleaved by ~ 200 ms) in favour of the single-NB firing regime (Fig 3E). Interestingly, we could mimic the superburst firing regime by including NMDA conductance in our model (Fig 3E, n = 10 simulations, AGN-networks). In the full model with the NMDA current, the ISI distribution of single spiking neurons was tri-modal (Fig 3F, black, n = 10 AGN-networks), whereas without NMDA currents (Fig 3F red, n = 10, AG-networks) it was bi-modal. The first peak (P1) corresponded to the mean firing within an NB and the second peak (P2) to the mean firing between consecutive NBs. The third peak was only obtained in AGN-networks (Fig 3F, highlighted with an arrow) and accounted for the time intervals between consecutive sequences of NBs in superbursts (see S6 Appendix for a detailed analysis of superbursts).

Overall, our results show that our computational model can express propagating NBs, as well as express different dynamics resulting from the manipulation of the main excitatory and inhibitory synaptic currents. Consistent with other previous studies [31,32,35,36,37], we found that AMPA currents drive NBs, while NMDA and GABA currents are only involved in shaping the dynamics of these coordinated spiking activity events. Following these results, the simulations in the next sections were performed with AG-networks endowed with AMPA and GABA conductance only. NMDA was not included because we found that its build-up during the initiation phase of an NB is much slower than the kinetics of AMPA (see Fig 3G).

### Local network properties at sites initiating coordinated spiking activity

**Fig 4:**
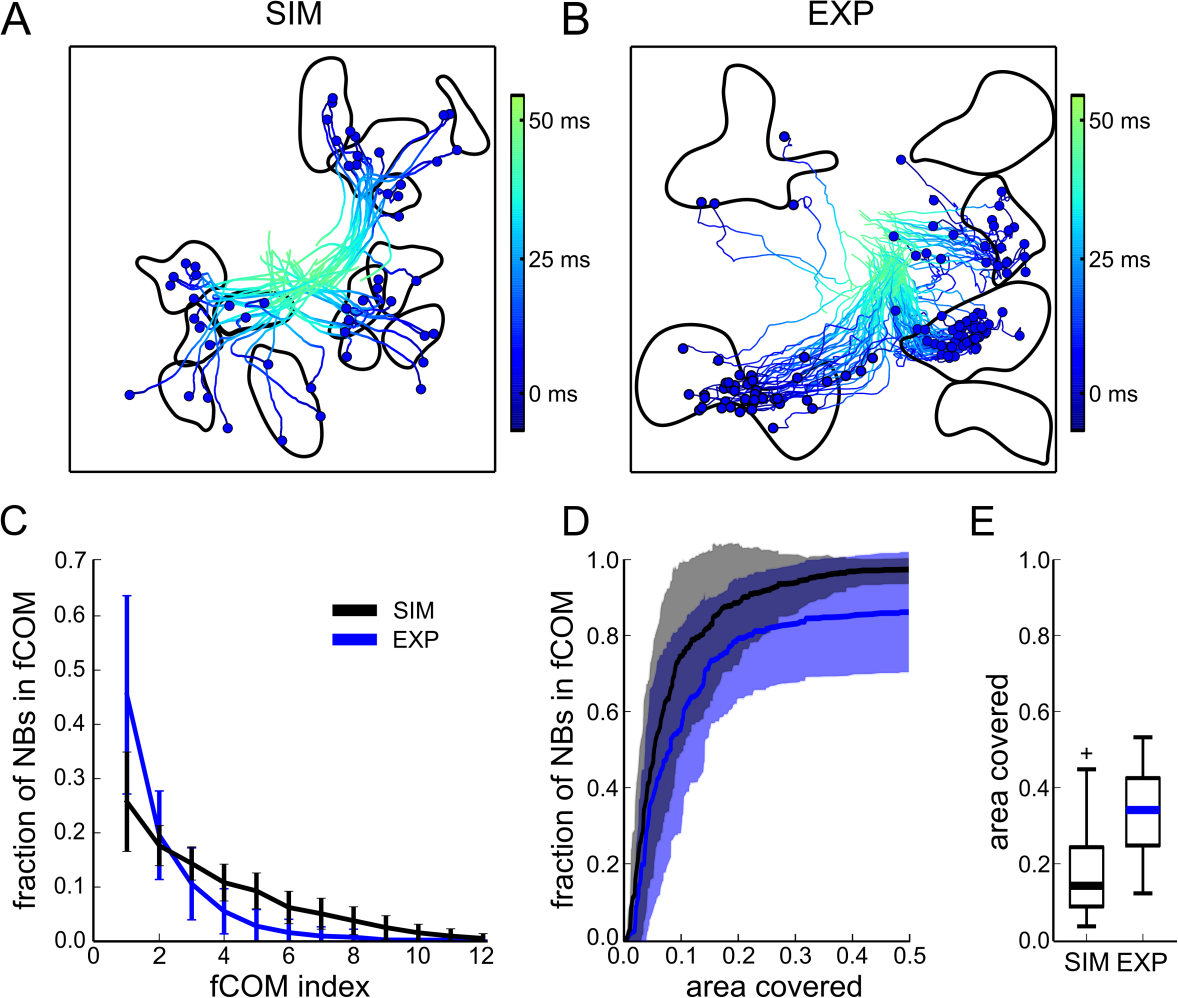
Network regions initiating spontaneous NBs correspond to functional communities. Spatial maps of the functional communities (fCOMs) and CATs (for clarity, CATs are only depicted up to 50 ms after NB initiation) computed for NBs in simulations (A) and recordings (B), show that the ignition sites (ISs, blue dots) of the NBs and the fCOMs (regions delimited by black solid curves) tend to overlap. A further quantification confirms that the overlap is statistically significant. (C) Fraction of NBs with ISs in a specific fCOM over the total number of NBs, for simulated (black) and experimental (blue) data. (D) Fraction of NBs with ISs located in an area defined by an increasing number of fCOMs (‘area covered’) over the total number of NBs (black: n = 20 simulations, blue: n = 15 recordings, mean value: solid line, standard deviation: shaded area). As shown, NBs originate from <50% of the network area. (E) Boxplots of simulated and experimental areas covered by fCOMs with respect to the total network area, showing that even though not all fCOMs are associated with an IS, the mean total area covered by all fCOMs is <40%.

Here, we investigated the properties of the network that might underlie the generation of NBs. To do so, we quantified the correlated spiking activity among pairs of neurons in both experimental and simulated data. Based on the obtained cross-correlation matrices, we built functional graphs, whose functional links connect highly correlated neurons (see Material and Methods). Interestingly, even though the cross-correlation does not explicitly takes into account the position of neurons (or of the electrodes), the functional connectivity analysis of both simulated and experimental data shows that these functional links tend to cluster into a few regions of the network (Fig 4A and 4B, see S7 Appendix for additional example). A further post-processing of the functional graph (i.e., with the Infomap algorithm) confirmed that these links clustered in a few (<10) spatially segregated regions of the network. In the following sections, we will refer to these regions of the network as functional communities (fCOMs).

Since in each dataset we have found a number of fCOMs regions that is similar to the one of the initiation sites (ISs) of NBs, we wondered whether the fCOMs regions might be associated with the initiation sites (ISs) of the NBs. As quantified in Fig 4C and 4D, we found that the position of the fCOMs co-localized with the ISs of the NBs and that the fCOMs covered a small area of the networks (Fig 4E). This overlap exceeded the 95% bootstrap threshold (see the Materials and Methods section) in 18 out of 20 simulated networks and in 10 out of 15 experimental recordings. We also observed the presence of fCOMs without any corresponding ISs (Fig 4B bottom-right), but all ISs of a cluster of NBs were always associated with a distinct fCOM. Therefore, these results indicate that the analysis of the fCOMs can be used to identify the potential regions of a network that initiate NBs. Indeed, the functional connections among neurons belonging to the same fCOM showed high cross-correlation peak values (0.52±0.06 and 0.19±0.04 for simulated and experimental data, respectively) and small time lags (less than 1ms, i.e., the neurons belonging to an fCOM fire almost synchronously).

**Fig 5:**
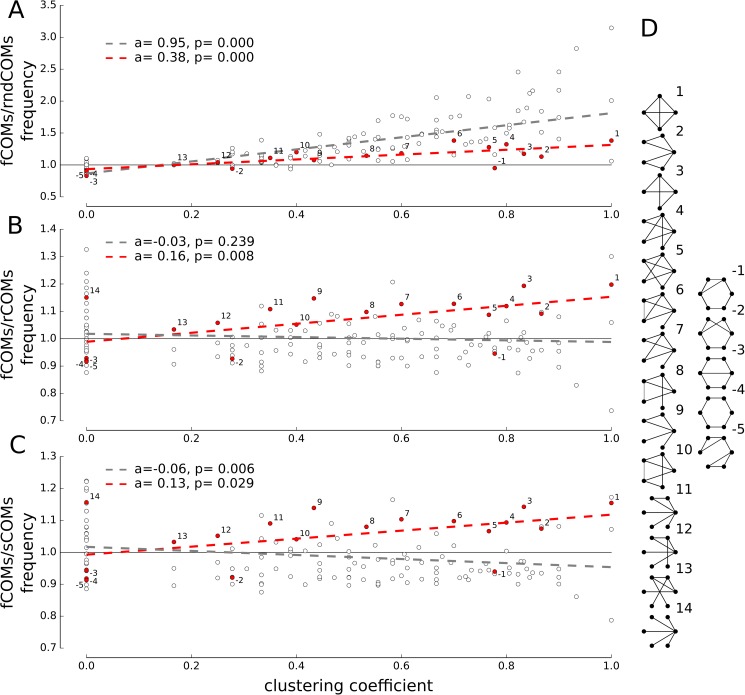
Analysis of the structural connectivity motifs in the functional communities of the network. Clustering coefficient of structural motifs tested (up to six nodes and isomorphic subgraphs) against the relative abundance of structural motifs in fCOMs and the null-models: rndCOMs (A), rCOMs (B) and sCOMs (C) (see Materials and Methods section). Red dots mark the structural motifs occurring with a significant frequency difference (p-value<0.05, t-test) for all null-models. Regression lines visualize the trend (where *a* and *p* denote the value of the slope and the p-value respectively) among all tested motifs (gray) and the statistically different ones (red). (D) Illustration of overabundant (positive index) and under-represented (negative indexes) structural motifs found in fCOMs with respect to the null models depicted in A-C. As highlighted by the red lines, a high clustering coefficient characterizes the structural motifs that are significantly over-expressed in fCOMs; conversely, significantly under-represented structural motifs resulted in low clustering coefficients (0.56±0.30 vs. 0.19±0.27, respectively).

These findings reveal that the fCOMs are associated with the ISs of the NBs and that the fCOMs show particular functional connectivity properties. This latter result suggests that the fCOMs (and, consequently, the ISs) may be formed by neurons with a particular local structural connectivity. Given that our simulation results have shown a good similarity with respect to experimental results (spontaneous activity (Fig 2G), pharmacological manipulations (Fig 3D and 3E) and on the fCOMs-ISs correspondence), thus validating the goodness of our model, we further exploited this network model to access experimentally hidden quantities. Specifically, we exploited the model to investigate the structural connectivity underlying the fCOMs. To do so, we quantified the occurrence of small template subgraphs, or structural motifs [38] (all possible non-isomorphic graphs up to six nodes, see the Materials and Methods section), in the structural connectivity underlying the fCOMs. In particular, we found a clear positive correlation (Fig 5A, gray line) between the clustering coefficient and relative abundance of motifs between the structural connectivity of fCOMs and rndCOMs (i.e. random graphs with the same number of edges, see Materials and Methods). To further test whether the clustering coefficient could be a key feature underlying the fCOMs in the anatomical network, we repeated the same quantification with respect to the rCOMs and sCOMs (i.e. graphs derived from the same network that generated the fCOMs but characterized by a high clustering coefficient, see Materials and Methods). Interestingly, although the clustering coefficient and the abundance of motifs were not anymore positively correlated in both rCOMs (Fig 5B, gray) and sCOMs (Fig 5C, gray), we found that a subset of all tested templates (Fig 5D) occurred preferably in the fCOMs. Among these motifs, the correlation between abundance and clustering coefficient (Fig 5A–5C, red dots), was always significantly positive (red lines, Fig 5A–5C), independently from the compared null model. In particular, the overabundant motifs in the fCOMs were characterized by a significantly higher number of recurrent connections (average clustering coefficient of 0.56±0.30, Fig 5D positive indexes), whereas the motifs underrepresented in the fCOMs were mainly composed of simple connectivity paths (average clustering coefficient of 0.19±0.27, Fig 5D negative indexes). This result was robust across different simulated networks (n = 20), suggesting that the high recurrence in the structural connectivity is a fundamental property of the regions eliciting NBs.

### Generation of network burst events in the functional neuronal communities

**Fig 6:**
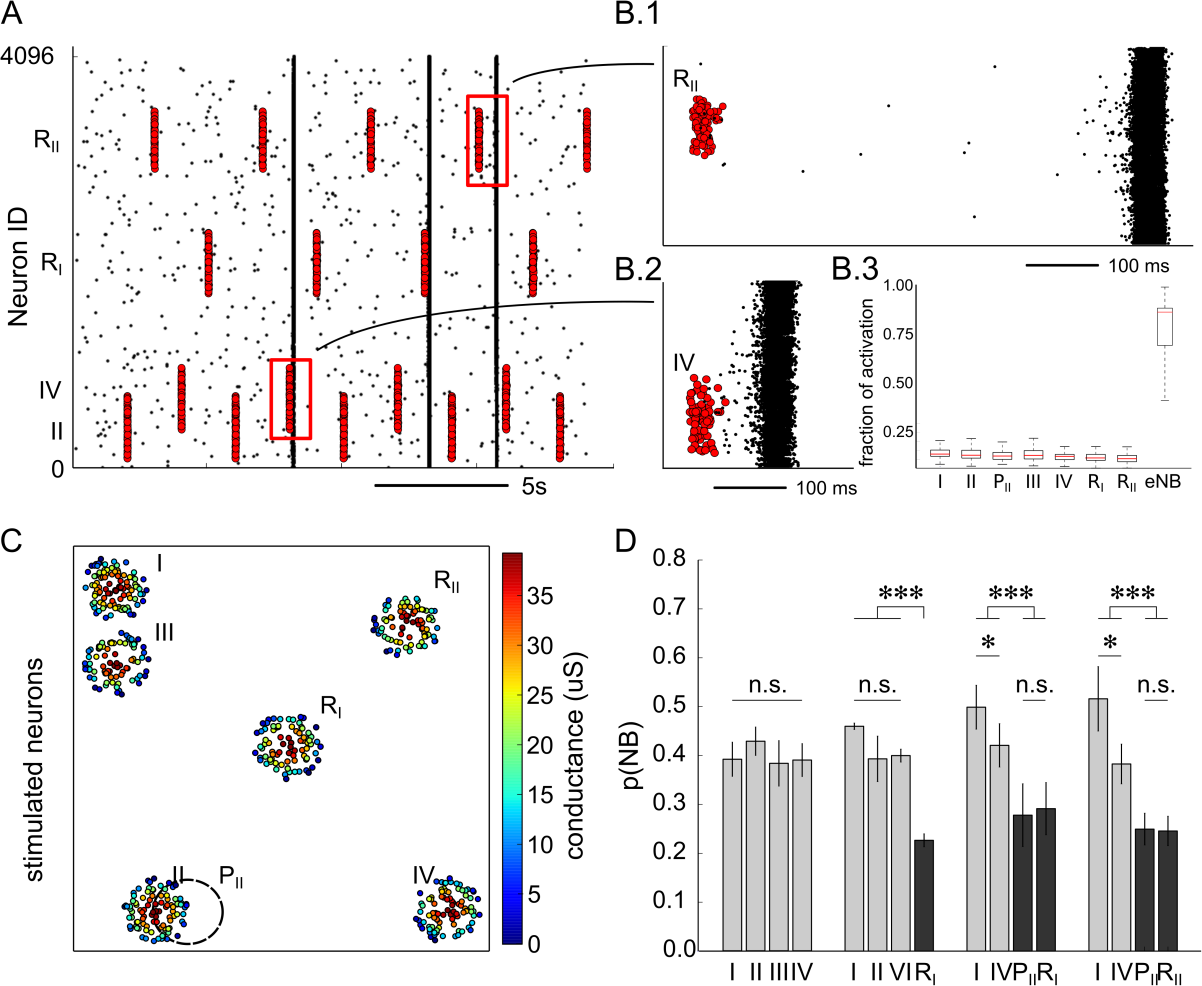
Ready-to-fire state of the functional communities. The fCOMs of the simulated networks (n = 10 networks simulations) were perturbed with mild-subthreshold stimuli to test their sensitivity in eliciting NBs (see the Materials and Methods section). (A) Raster plot of the activity (black dots) with overlaid electrical stimuli (red dots). As shown, stimuli can either marginally affect the spontaneous activity (B.1) or evoke an NB (B.2). When an NB is elicited, almost all of the stimulated neurons fire (B.3, eNB) a spike within 50 ms. In contrast, when an NB is not elicited, only a small fraction of stimulated neurons fire a spike. In this case, the neuronal activity is not significantly different between the probed regions. (C) Representation of the spatial arrangement of the fCOMs and reference regions that were probed in this example. Neurons are colour coded according to the strength of the stimulus. Stimuli are delivered to fCOMs I, II, III and IV (c.f. Fig 4A) and to reference regions R_I_, R_II_, P_II_. (D) The perturbation of the fCOMs evokes NBs with a higher probability than the reference sites.

Next, we investigated possible mechanisms of NB generation associated with the structural properties of the fCOMs. Indeed, these regions may act as local amplifiers of spontaneously elicited spikes (i.e., Poisson spikes) or fading activities deriving from previous activation of the network. We tested this hypothesis of local amplification in our model by perturbing regions of the network with mild subthreshold stimulations (Fig 6A). Importantly, to assess differences among different network regions, we used a mild perturbation that did not ensure the generation of an NB, but rather, could be ineffective (Fig 6B.1) or effective (Fig 6B.2) at generating an NB. As shown in Fig 6B.3, the selected stimulation amplitude gave rise either to a small or large fraction of spiking neurons depending on the effective initiation of an NB but independent of whether the region was an fCOM.

In a representative case (see Fig 6C), we stimulated seven regions of the network described in Fig 4A. These regions consisted of four fCOMs (i.e., *I*, *II*, *III and IV*) and three reference regions (*R*_*I*_, *R*_*II*_, *P*_*II*_*)*. The latter reference regions were chosen to test the effectiveness of a perturbation when delivered to the center, the border of the network (*R*_*I*_ versus *R*_*II*_) or to a shifted position in an fCOM (*P*_*II*_ versus *II*). Each protocol of stimulation consisted in the perturbation of four target regions (I-II-III-IV), (I-II-IV-R_II_), (I-IV-P_II_-R_I_) and (I-IV-P_II_-R_II_) as depicted in Fig 6D. The simulation results show that the subthreshold stimulation delivered to the fCOMs had a significantly higher probability of evoking NBs than stimulations delivered to reference regions of the network (Fig 6D). Moreover, as shown in the figure, the results indicate that to evoke NBs reliably, the stimulation has to be focused in the centre of the fCOMs (the probability of evoking an NB by confining the stimulation to *II* is significantly higher than by delivering it to *P*_*II*_, *R*_*I*_ and *R*_*II*_). Therefore, fCOMs are spatially selective to subthreshold stimulations. On the other hand, the number of reference regions probed can enhance the probability of evoking NBs in fCOMs (c.f.r. I and IV across different paradigms of stimulation). Altogether, these results indicate that fCOMs are specialized regions of the network that can initiate NBs by locally amplifying background spiking activity.

### Spiking activity patterns anticipating network bursts in the functional communities

**Fig 7:**
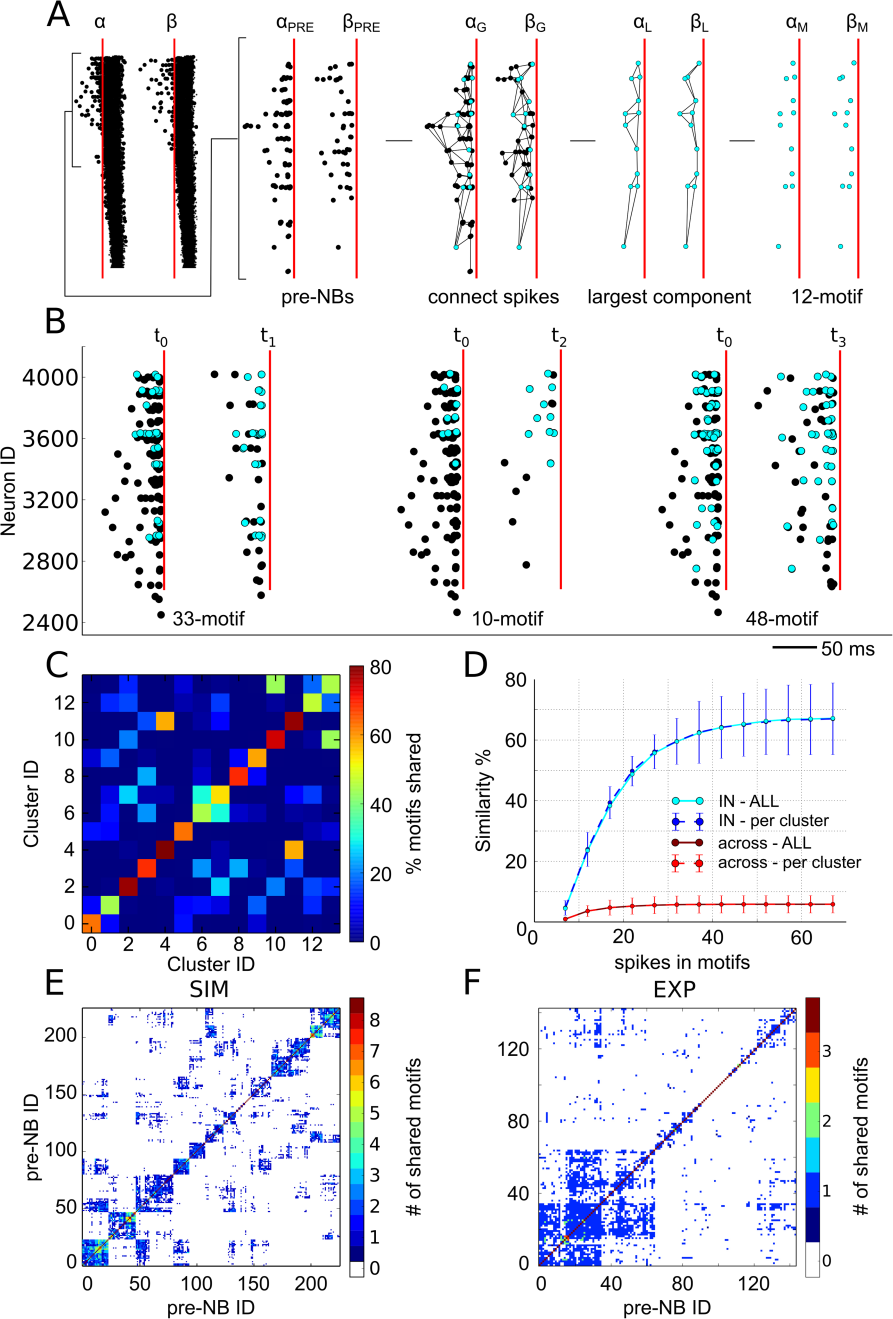
The pre-NB activity shares similar temporal motifs for NBs in the same class and is informative of the following coordinated event. (A) Illustrative representation of the analysis chain used for the pre-NB temporal motifs, where we considered the following: two NBs (*α*,*β*), their pre-NB activity (*α*_*PRE*_,*β*_*PRE*_), the NB-graphs (*α*_*G*_,*β*_*G*_), the largest connected components (*α*_*L*_,*β*_*L*_) and the corresponding number of spikes in the shared temporal motifs (*α*_*M*_,*β*_*M*_, M = 12 spikes). (B) All NBs of the same cluster (e.g., cluster ID 0) share common pre-NB temporal motifs (cyan spikes). For instance, the NB occurring at time *t*_0_ shares a temporal motif with the event at time *t*_1_ but differs from the temporal motif shared between NBs occurring at time *t*_2_ and *t*_3_. (C) Normalized similarity matrix among pre-NBs activities (M-motif, with M > 5) for clustered NBs. The clustered NBs share the highest number of motifs (i.e., higher values on the diagonal), indicating that the pre-NB activity is informative of the following NB. (D) Cumulative similarity plot corresponding to the data in panel C but normalized with respect to the cluster size (per-cluster, dashed line) or to the total number of NBs (solid lines). The similarity is consistently higher for the pre-NB activity of NBs belonging to the same cluster (IN) than for the NBs of other clusters (ACROSS). (E) Reordered similarity matrix of pre-NB activity of a simulated network, showing a block structure. Each block is relative to an NB cluster characterized by similar pre-NB spiking patterns. (F) Reordered similarity matrix of recorded pre-NB activity in a cultured network, still shows a block structure.

Finally, we investigated whether the spiking activity preceding an NB (or pre-NB spikes) could be predictive of the following NB, as previously suggested [39]. To compare the pre-NB spiking activity of different coordinated events, we isolated the largest spiking pattern (see the Materials and Methods section) shared by the pre-NB activity of each pair of NBs (Fig 7A, number of shared spikes M = 12). Illustrative examples of these temporal motifs shared by the pre-NB spiking activity of events in an NB cluster are reported in Fig 7B (see S8 Appendix for additional examples).

We found that the similarity measure of the temporal motifs (see the Materials and Methods section) computed for the pre-NB activity of an NB cluster was significantly higher than for the pre-NB activity of NBs belonging to different clusters (Fig 7C). The similarity measure increased with the maximal size of the temporal motifs and reached a plateau at approximately M = 50 spikes (Fig 7D). While most of the pre-NBs activities shared only from M = 6 to M = 20 spikes, some of them shared more than M = 40–50 spikes, suggesting that NBs might need the coordination of a different number of spiking neurons for their generation. Given the similarity of the pre-NB activities of events belonging to the same NB cluster, we used this measure of similarity to cluster the NBs rather than using the analysis of the CATs. We found that upon reordering the NB events with a hierarchical clustering algorithm and considering pre-NB activities (Fig 7E), the similarity matrix exhibited a block structure similar to the one obtained with the clustering of the CATs (c.f. Fig 2E). Next, to verify these computational findings in biological neuronal networks, we investigated whether stereotyped pre-NBs activities also characterize the initiation phase of NBs in our recordings. Consequently, we computed the similarity matrix of pre-NB activities on experimental recordings and, although less pronounced than in simulations (Fig 7F), we also obtained a block structure similar to that obtained from the simulated activity (Fig 7E). In summary, these results, obtained by simulation and verified experimentally, suggest that NBs are not induced by random spiking activity patterns in the fCOMs, but are rather induced by similar patterns of pre-NB spikes.

## Discussion

While changes in the dynamics of coordinated, spontaneous spiking activity have been investigated in different *in vivo* and *in vitro* neuronal systems, the mechanisms underlying the generation of these propagating waves of spikes still remain unclear. In this work, we investigated the generation of these events, or NBs, in spontaneously active neuronal cultures by exploiting high-resolution multielectrode array recordings, computational network modelling and the reduced complexity of these isolated 2D network models. As shown in our experimental results and in accordance with previous studies [15,18,37], a cultured network expresses only a few classes of NBs, each one having a distinct and local IS. We found that the initiation sites of NBs correspond to the fCOMs of neurons where the strongest neuronal functional links of the network are clustered. The analysis of the structural connectivity in the computational model reveals that these regions initiating NBs in the network share similar connectivity motifs and have a higher level of recurrent local connections with respect to other similar regions of the network. We also show that the local connectivity properties of the fCOMs may enable these regions to act as amplifiers of the pre-NB spiking activity and that this property is most likely the underlying mechanism generating NBs. Indeed, when stimulated with sub-threshold stimuli, the fCOMs show a higher probability of generating an NB than any other equivalent sub-region of the network. Consequently, the fCOMs may initiate NBs by amplifying local asynchronous spiking activity, a concept similar to the ‘noise-focusing’ proposed in [19]. Interestingly, previous works [21,39] have indicated that NBs are preceded by the activation of a subset of overactive electrodes. As suggested by our simulations and confirmed with experimental data, our analysis of the pre-NB activity shows that NBs of the same class (sharing a similar propagation trajectory and initiation site) are indeed triggered by pre-NBs spiking patterns sharing similar temporal motifs. These patterns of spiking activity preceding NBs of the same class are not only spatially confined to the surround of the sites initiating an NB but also determine the following NB propagation.

To perform this study, we developed a computational network model able to both qualitatively and quantitatively replicate the spontaneous network activity recorded in our experiments. This allowed us to study the properties of the regions initiating NBs with full access to the structural and functional neuronal connectivity. Previous computational modelling studies on cultured networks have shown that NBs can be robustly replicated using different cellular, synaptic and network settings [14,30,40,41,42,43]. However, none of these computational network models displayed spontaneously propagating NBs as obtained in our model. In our work, the simulated spontaneous activity of each neuron is induced by independent subthreshold synaptic inputs whose occurrence is modelled by a Poisson process. However, this process gives rise only to sparse single-neuron spikes in the network and it is not sufficient for the generation of coordinated spiking events. Here, we have exploited our model to investigate what allows a network to turn this sparse activity into travelling waves of spikes and to explain why NBs are always elicited from specific regions of the network as observed experimentally. Notably, by adopting rather simple and biologically plausible connectivity rules (i.e., the connectivity probability among neuron pairs decays with the relative distance), we found that the model is able to: i) spontaneously express a few classes of propagating NBs as observed in *in vitro* experiments, ii) fire asynchronous and synchronous spiking activities with statistical properties close to those of experimental recordings, and iii) recapitulate previously reported experimental findings on cultures manipulated with AMPA, GABA and NMDA pharmacological blockers.

The validation of the model against pharmacological manipulations also allowed us to evaluate the contribution of AMPA, GABA and NMDA synaptic currents in shaping the NB dynamics. In accordance with previously reported results, our data show that AMPA is required to drive the network from uncoordinated to coordinated firing regimes, while NMDA and GABA synaptic currents are mainly involved in shaping the NB dynamics. The inhibitory GABA current mainly regulates the duration, strength and frequency of NB occurrences. Interestingly, the shorter NB duration in the GABA-OFF condition, observed in both the *in vitro* experiments [17] and in the model, suggests that inhibition keeps the firing rate low and prevents a strong depression of the excitatory synapses. The excitatory NMDA, in turn, is a key player for the insurgence of the superburst firing regime. Our model suggests that the slow dynamics of the NMDA currents counteract synaptic depression and neuronal adaptation, indicating that the closure of a superburst event occurs when the latter two mechanisms prevail over the NMDA current. These results show, similar to previously reported *in vivo* [44] and *in vitro* [45] experimental data, that the NMDA current can sustain persistent activities in the network.

Overall, our results suggest that the generation of events of coordinated spontaneous activity in a neuronal system might be related to the presence of fCOMs rising from the local and inhomogeneous connectivity of neurons in the network. This finding suggests that for a developing neural system that is able to express coordinated spontaneous activity, parallel to reshaping the cellular and synaptic properties [46], forming regions in the network with local heterogeneities (such as neuronal density or neuronal processes density) that can establish a high degree of local recurrent structural connections might be very important. However, our considerations are derived from the study of *in vitro* neuronal cultures that certainly show a simpler organization compared to that of brain circuits developing in 3D. Given that 3D neuronal cultures have been shown to display spontaneous NBs [47,48] and considering that a 3D implementation of our model has been shown to allow for the interpretation of distinct spiking activity regimes characterizing 2D and 3D experimental preparations [47], the generation of NBs in a 3D neuronal network might be investigated in future works. The results of the present study allow us to suggest that fCOMs of neurons may naturally emerge by following simple constraints of distance-based connectivity. However, to address this hypothesis with simulations, a probabilistic growth model [49] describing the critical parameters during the process of network formation should be developed in future work. Finally, the presented network model might be directly applicable to the interpretation of experimental recordings of spontaneous activity changes induced by neuroactive compounds or may provide complementary information on the integration at the cellular scale of electrical [50] or optogenetic [51] external stimuli.

## Materials and methods

### Ethics statement

All procedures involving experimental animals were approved by the institutional IIT Ethic Committee and by the Italian Ministry of Health and Animal Care (Authorization number 110/2014-PR, December 19, 2014).

### Large-scale recording of neuronal spiking activity in cultured networks

#### Cell cultures and high-resolution CMOS-MEA recordings

Primary hippocampal neurons from rat embryos (at embryonic day 18, E18) were dissociated following procedures as described in [22,52] and plated on CMOS multielectrode arrays (CMOS-MEAs, Biochip 4096E, from 3Brain GmbH). Chips were previously sterilized with 70% ethanol, conditioned overnight in an incubator with cell culture media and coated with adhesion-promoting molecules, i.e., a double layer of 0.1 mg/ml poly-L-lysine (Sigma P-6407) and 0.1 mg/ml laminin (Sigma L-2020). A few hours after plating at a nominal cellular density of approximately 3000 cell/mm^2^, the cell culture reservoir of each device was filled with 1.5 mL of Neurobasal cell culture media (Thermo Fisher, #21103049) supplemented with B-27 (Thermo Fisher, #17504044) and placed in a humidified incubator (5% CO2) at 37°C. Cell cultures were grown on a chip for 19–21 days *in vitro*, an age where sustained spontaneous electrical activity characterized by single spikes and short bursts propagating through the network is observed.

The extracellular activity of the cultures was recorded from 4096 electrodes for 10 minutes using a custom recording system similar to the BioCam platform commercially distributed by 3Brain GmbH. The electrode array provides 4096 square electrodes (21 x 21 μm^2^, 82 μm electrode pitch) covering an active area of approximately 5 x 5 mm^2^. Pharmacologically manipulated activity with bicuculline (BIC) at 30 μM or (2R)-amino-5-phosphonovaleric acid (APV) at 50 μM was also recorded for some cultures after adding the compound to the cell culture media. All the raw data were stored as .brw files (BrainWave, 3Brain GmbH) and then exported to Python (Python Software Foundation, Python Language Reference, version 2.7.) for further analysis (c.f. “Data analysis of experimental and simulated data”). The spike trains of neuronal recordings are available at doi:10.5061/dryad.5k67r.

### Computational network model

#### Spiking neuron model

The computational network model is composed of a set of excitatory and inhibitory spiking neuronal models implemented in NEURON [53]. We used the Adaptive Exponential Integrate and Fire (AdExp) neuron model described in [25], which, similar to the Izhikevich model [54], represents a good compromise between computational costs and the capability of mimicking the variety of firing patterns exhibited by real neurons. The differential equations governing the AdExp dynamics are as follows:

$$\left\{ \begin{array}{c} C\frac{dV}{dt}=-g_{L}(V-E_{L})+g_{L}\Delta Te^{\left(\frac{V-V_{T}}{\Delta T}\right)}-w+\sum I_{syn}+I_{bg}\\ \tau_{w}\frac{dV}{dt}=a(V-E_{L})-w \end{array}\right.$$

The variable *V* represents the membrane potential, and *w* is an internal state variable responsible for any adaptive phenomena. The voltage *V* is governed by a leak current (conductance *g*_*L*_, reversal potential *E*_*L*_), a *Na*^+^ -like current involved in the upswing of the action potential given by the exponential term, an adaptive current *w*, the synaptic currents *I*_*syn*_ and a background noise *I*_*bg*_ current. The adaptive current *w* is modulated by the voltage and relaxes back to its equilibrium with the adaptation time constant *τ*_*w*_. Regarding the spiking mechanisms, whenever the voltage crosses the threshold of 0 mV, a spike is emitted, and the state variables are reset (*V* → *V*_*reset*_, *w* → *w* + *b*). In our model, the parameter settings of the AdExp were adapted from [55]. Because neurons do not fire isolated bursts at the mature stage of cell culture [11], we assumed standard spiking models for the excitatory and inhibitory neurons [30] with the same 4:1 ratio. Excitatory neurons were modelled as characteristic adaptive firing neurons, and inhibitory neurons mimicked the firing of fast-spiking interneurons. Then, to consider the heterogeneity of cells in neural cultures and to ensure that network synchronization was not a consequence of identical properties of single cells [40,14], the parameters of the modelled neurons were drawn from a normal distribution (see S2 Table for values).

### Synaptic communication

Previous studies highlighted that the coordinated activities in cell cultures are determined by the chemical synapses and not by gap junctions or extracellular substances [56]. Therefore, we modelled the dynamics of excitatory AMPA and NMDA as well as inhibitory GABA chemical synapses. Synaptic transmission was delayed by a fixed time (0.5 *ms*) to account for synapse activation and a variable delay (maximum 1.5 *ms*) to account for the propagation of the pre-synaptic spike. Each type of synapse contributed with a current *I*_*syn*_ modelled as follows:

$$\left\{ \begin{array}{c} I_{syn}=g_{syn}(v-E_{rev})\\ \tau_{syn}\frac{dg_{syn}}{dt}=-g_{syn} \end{array}\right.$$

where *g*_*syn*_ is the synaptic conductance and *E*_*rev*_ is its reversal potential. The synaptic conductance has a bi-exponential profile (parameters in S3 Table):

$$\left\{ \begin{array}{c} \tau_{syn}\frac{dg_{syn}}{dt}=-g_{syn}\\ \tau_{rise}\frac{dg_{rise}}{dt}=-g_{rise}\\ I_{syn}=(g_{syn}-g_{rise})(v-E_{rev}) \end{array}\right.$$

Each time an action potential is delivered to a target neuron (i.e., a time *t*_*sp*_), the conductance parameters *g*_*syn*_ and *g*_*rise*_ are increased by $\bar{g}\times y$, where $\bar{g}$ is the maximum value for the synaptic conductance and *y* is the fraction of the active resources (i.e., released neurotransmitters). The synaptic current exhibits short-term depression modelled under the assumption of finite synaptic resources [57]:

$$\left\{ \begin{array}{c} \frac{dx}{dt}=\frac{z}{\tau_{rec}}-u\cdot x\cdot \delta (t-t_{sp})\\ \frac{dy}{dt}=-\frac{y}{\tau_{1}}+u\cdot x\cdot \delta (t-t_{sp})\\ \frac{dz}{dt}=\frac{y}{\tau_{1}}-\frac{z}{\tau_{rec}} \end{array}\right.$$

where *x*, y and *z* represent the fraction of available, active and recovered resources, respectively. The time constant *τ*_1_ regulates the transition between the available and active state, *τ*_*rec*_ is the recovery time constant and *u* represents the fraction of available resources transferred to the active ones, when the synapse is activated.

The NMDA current was modelled similarly to the AMPA and GABA currents, with an additional magnesium block mechanism [58]:

$$I_{NMDA}=g_{NMDA}\cdot (v-E_{rev})\cdot 1/\left(1+exp(-(v-v_{0})/k_{0})\right)$$

where *k*_0_ = 6 mV (steepness of voltage dependence) and *v*_0_ = −40 mV (half-activation potential). The maximum NMDA conductance (${\bar{g}}_{NMDA}$) is written in terms of the AMPA conductance: ${\bar{g}}_{NMDA}=K_{NMDA}\cdot {\bar{g}}_{AMPA}$ such that in basal/standard conditions *K*_*NMDA*_ = 0.09 (i.e., ${\bar{g}}_{NMDA}$ = 4.32 *nS*), and while under APV application, an NMDA antagonist, *K*_*NMDA*_ = 0. All parameter values are reported in S3 Table. To mimic the effects of the NMDA and GABA synaptic blockers (APV and BIC), the conductance of the target receptor was set to zero. Networks with only the AMPA and GABA synapses are called AG-networks in the text, and networks with in addition the NMDA current are called AGN-networks.

### Background activity of the network

From its earliest days *in vitro*, cultured neuronal networks display random spontaneous spiking activity. In the model, this activity was mimicked by injecting sub-threshold synaptic noise (i.e., miniature events [56]) modelled as independent Poisson processes at a mean frequency of 25 *Hz*. The summation of the synaptic noise occasionally brought the neurons to fire in the uncoupled network and it determined a background spiking activity of 0.010 ± 0.007 *Hz* (close to the reduction of spiking activity found in experiments when AMPA receptors are blocked with CNQX [31,33]).

### Network topology

Although network topology has been recognized to play an important role in determining network activity, many works have neglected the spatial constraints derived from the location of neurons in the network [42,43] (see also S1 Appendix). To be comparable with our experimental recordings, 4096 neurons were uniformly distributed on a unit square, and the connectivity probability among the neurons depended on the distance according to a radial Gaussian function. The distance-based connectivity rule allowed for the creation of biologically inspired networks whose graph properties (i.e., clustering coefficient or the presence of shortcuts) were not imposed but were rather inherited from the imposed spatial organization of neurons. Only graphs without any isolated component were used in the simulations. It is important to highlight that the random arrangement of neurons, the distance-based connectivity rule and the sparseness of the connections used to establish the network topology gives rise to an inhomogeneously connected network that includes clusters of nodes with denser connections (see S2 Appendix for additional details). As a consequence, although the node degree is quite comparable between the Gauss and random graph, the clustering coefficient of the former is significantly higher than in the latter one, (see S3 Appendix for additional details on clustering coefficient, link length and shortest path length [38]).

The directionality of the synaptic connections between pairs of neurons was assigned with equal probability. Although bidirectional connections are quite common in the brain, computer simulations have shown that networks with only depressing synapses (as assumed in this work) tend to evolve unidirectional connections [59]. Regarding the connectivity of the network, in 2D cell cultures, each neuron receives somewhere from 150 to 400 synapses [60]. Because each neuron is contacted by eight synapses from the same neuron on average [61], the actual effective synapses in the model were decreased to 41.6 ± 6.4 synapses per neuron. S1 Table summarizes the graph properties (e.g., clustering coefficient, mean path length, degree [62]) of the simulated network. Note that neurons at the border of the domain were treated exactly like the other nodes and were consequently connected to a smaller number of neurons. The network model is available at doi:10.5061/dryad.5k67r.

### Stimulation of ignition sites

A stimulation protocol was designed to probe the sensitivity of the modelled network to respond to local stimulations. To this aim, mild sub-threshold stimuli were delivered to specific sub-regions of the network (e.g., *R*_*I*_ Fig 6C) composed of ≃ 40 neurons. The stimulation consisted of Poisson spike trains at 1 Hz (per neuron) that activated currents with the same time course of AMPA receptors. In addition, to ensure that the stimulation was effectively confined to local sub-regions of the network, the conductance was imposed to decay from the centre of the sub-regions. That is, for $d\leq 0.08,\;\left(1-(\frac{d^{2}}{0.08})\right)\;g_{ST}\;with\;g_{ST}=45nS$ and for *d* > 0.08, the conductance was *g*_*ST*_ = 0 *nS*. As depicted in Fig 6D, each stimulation protocol involved four different sub-regions of the network (100 stimuli per sub-region, maximum two non-fCOMs sub-regions), which were alternated with a pseudo-random sequence to minimize interferences among subsequent stimuli. The rationale for this choice was to probe reference regions of the network while continuously monitoring the success rate of eliciting NBs by stimulating the fCOMs. NBs occurring later than 150 ms from the stimulation were counted as spontaneous activity.

### Data analysis of experimental and simulated data

To facilitate the comparison with the experimental data, the simulated spike trains were subjected to the same filtering criteria used in the experiments. Thus, only neurons whose firing rate (i.e., average number of spikes per unit time) fell in the interval [0.1–15] Hz were considered for all subsequent analysis. In the manuscript, significant differences among normally distributed groups were evaluated through an independent or paired t-test, while a Mann-Whitney’s U test and Kolmogorov-Smirnov test were used for non-normally distributed samples. In the text and figures, the reported error bars are standard deviations.

### Detection and quantification of network activity

#### Spike detection and spike-based quantification

We quantified the spiking network activity by using standard activity parameters [31,50] such as the mean firing rate of the network (MFR) and the inter spike interval (ISI) distribution. The MFR is the average of the firing rates of all of the active neurons of the network, and the ISI is the first order difference of the spike times. Additionally, to characterize the network burst regime, we quantified canonical parameters such as the mean bursting rate (MBR), the mean firing intra burst (MFIB) and the mean burst duration (MBD), as in [63]. Finally, as most of the neurons in these networks participate in NBs with a burst of spikes, we defined an indirect measure of asynchronous network activity (Random Spikes), as the percentage of spikes that are not part of a burst (a sequence of 5 or more spikes separated by less than 100 ms). Bursting events (NBs) are stereotyped network activities characterized by a large fraction of neurons simultaneously active for ≃ 100*ms*, and thus, these events could be detected by setting a hard threshold on the instantaneous MFR [15,39]. This algorithm works well on simulated data, but on real experimental data, the detection of NBs can be hindered by noise (e.g., false-positive spikes). We have therefore designed an alternative algorithm (NB-graph) based on a graph theory approach that overcomes this limitation. A detailed description of the algorithm and a comparison with the standard procedure used to detect NBs is reported in S4 Appendix.

#### Spatial and temporal profile of the network bursts

The temporal and spatial resolution of our data allowed for the faithful investigation of the dynamics of the network bursts, particularly how NBs occur over time and if they share some similarity that could be explained from the underlying organization of the network. To this end, we computed the NB correlation matrix to study groups of neurons with similar firing patterns. The entries ${\bar{C}}_{n,m}$ of the NB correlation matrix [37,64] were given by the following equation:

$${\bar{C}}_{n,m}=\underset{\tau }{max}\left(\sum_{i=1}^{N}C_{n,m}^{i}(\tau )\right)$$

where the sum runs over the neurons, and the maximum is taken on the time window of the event (e.g., 0 < *τ* < 150*ms*). The term $C_{n,m}^{i}(\tau )$ represents the cross-correlation between the NBs *m* and *n* of neuron *i*. Such entries are then reordered using a standard hierarchical clustering algorithm aimed at highlighting the presence of similar NBs. The optimal cut point of the dendrogram was obtained by maximizing the Silhouette score. In addition, the spatial propagation of the spiking activity during an NB was also represented in terms of its centre-of-activity trajectory (CAT, [26,18]).

The CAT collapses the overall network activity to its centre of mass (i.e., regions of the network with more activity have a higher weight), allowing for the representation of how the activity in the network evolves over time with just two coordinates and the clustering of NBs with similar propagation trajectories. In our analysis, at each time point, the CAT was computed over 20-ms time bins with a sliding of 1 ms. To cluster CATs with different durations (e.g., when inhibition is blocked), the NBs were realigned to a common time interval.

### Network dynamic analysis

Emergent network activity can be explained to a large extent by the anatomical connectivity [38]. However, such activity can also be determined by the particular dynamical state of the network. Thus, an analysis of the statistical relationships between firing neurons can be informative of the information flow in the network. To determine the strength of the functional connections in the network, we performed a cross-correlation analysis [30]. Functional links were selected to meet two requirements. First, we considered the pairs of neurons whose cross-correlation peak was above the 95^th^ percentile of all the computed cross-correlation values. Second, for each selected pair, we assigned a functional link every time that their cross-correlation peak was ranked in the top of the ten strongest correlation peak values for both neurons, thus determining a bi-directional relationship. The first condition avoids the inclusion of spurious functional connections in the analysis. The second condition reveals potential structural network motifs that determine synchronous activities in the network. The bi-directional functional connections clearly do not correspond to structural ones (that are unidirectional, see Section Network Topology in the Materials and Methods section). Indeed, the functional graphs shared only 4.9 ± 1.8% of connections with the anatomical graph. However, these conditions allow for the determination of pathways of activity of neuronal pairs that receive similar inputs, either direct or indirect, from common firing neurons. The functional links with the longest connection (longer than 0.2, which roughly corresponds to 550 *μm* on the CMOS-MEA) were discarded from the analysis.

#### Detection of ignition sites

An additional measure was used to analyse subgraphs characterized by a strong level of internal connectivity (also referred to as community structures [65]). Ideally, the network is divided into groups of nodes with a maximally possible number of within-group links and a minimally possible number of between-group links [65]. We estimated communities through the Infomap approach [66], which determines subgraphs in a given network by minimizing the expected length of random walks over possible network partitions. To test the reliability of the procedure, we validated our results by varying the topologies (i.e., random, radius and Gauss graphs) as well as by changing the neuronal connectivity within the network (n = 10).

The overlap between the ignition sites (ISs) and the fCOMs was quantified as follows. First, we defined the area covered by an fCOM as the concave hull defined by the set of neurons of the fCOM. Second, to address border effect problems (i.e., assign the events that start close to the border of an fCOM to that fCOM), we extended the confines of the fCOMs by a factor of 5%. To assess if the ISs significantly overlapped with the fCOMs, we randomly reassigned (500 times) the detected fCOMs to another position in the network and quantified the overlap.

#### Quantification of structural connectivity motifs

To quantify the occurrence of small template subgraphs, i.e., structural motifs, in a given region of the network, we proceeded as follow. First, we considered all possible connected graphs of less than six nodes and built a list of motifs to test. Second, for each motif, we determined the number of isomorphic subgraphs in the target graph [67]. Finally, those numbers were normalized to the total motifs found in the target graph. Due to computational limitations for this extensive research, graphs had to be turned into their undirected counterpart. To assess significant differences in the motif composition of a given subgraph (fCOM), we defined three different null models (sCOMs, rCOMs, and rndCOMs) with the same number of nodes as that of the original subgraph. The sCOM was generated following a two-step procedure. At first, we applied the Infomap algorithm to a portion of the structural network that excluded the nodes of the fCOMs. The Infomap algorithm [65] allows for the partitioning of the complementary network into structural communities for which the information flow within the community is maximized and the information transmission towards the remaining neurons of the network is minimized. To faithfully compare an sCOM to an fCOM, the size of the sCOM was constrained to be the same as that of the fCOM. This down-sampling was performed by removing nodes from the sCOM and maximizing the spatial density of the remaining nodes of the subgraph. The latter step was intended to avoid poorly connected regions of the network.

The rCOM subgraphs were obtained as follows. First, a node in the complementary network was randomly chosen, and the closest K-1 nodes were aggregated (K is the size of the compared graph).

Finally, the rndCOM subgraphs were obtained by turning the original fCOM into a random graph, i.e., preserving the number of nodes and edges but randomly reassigning the links.

Even though the sCOMs and rCOMs have a different number of edges than the fCOMs, they are useful to compare equivalent regions of the network that do not elicit NBs. However, the rndCOMs have the same number of edges as the structural graph and can be used as a null model to investigate the relevance of the topology. The significant difference in the motif composition was assessed through paired t-tests (fCOMs compared to null models) at level p = 0.05.

#### Detection and clustering of pre-NB spiking activity

To relate the spiking activity preceding a spontaneous burst event (pre-NBs) to the subsequent propagating event, we took advantage of the NB-graph algorithm (see S4 Appendix, with parameters τ_NB_ = 10 ms, d_NB_ = 1/8). With the aim of determining whether pre-NB spikes shared similar spatiotemporal structures (i.e., temporal motifs), all pre-NB spikes falling in the 100 ms preceding the starting point of an NB were analysed. This was done by considering the largest connected component (i.e., α_L_, β_L_ of Fig 7A) of the induced NB-graphs (i.e., α_G_, β_G_ of Fig 7A). Two pre-NB motifs that share a common subgraph of at least M = 6 spikes (6-motif) are declared as similar (e.g., Fig 7B). Importantly, the parameters τ_NB_, and d_NB_ allow for the declaration of two spike patterns as being similar even if they are not identical. That is, shared temporal patterns can be regarded as jittered versions of the same spike pattern, and the similarity measure is robust to these variants.

The similarity of the network motifs was computed in terms of the matrix: *SM*(*X*,*Y*) = ∑_*x*_∑_*y*_ *K* (*x*,*y*)/*N*, where x,y are NB events of the clusters X,Y and the sums run over the x,y elements of the clusters X,Y. K(x,y) is the Kronecker distance (equal to 1 if x is similar to y, 0 otherwise), and *N* is a normalization factor given by *N* = ∑_*y*_ *S M* (*X*,*y*) ∙ ∑_*x*_ *S M* (*x*,*Y*). To compare the pre-NB spiking sequences for all NBs, a similarity index was introduced and defined as follows:

$$S=\frac{\sum_{X}\;S\;M(X,X)}{\sum_{X,Y}\;S\;M(X,Y)}$$

To quantify the similarity among NBs, we defined the ‘per-cluster’ measure as a weighted average with respect to the cluster size and the ‘ALL’ measure, defined on all trajectories irrespective of the cluster’s size. To further characterize how the number of shared pre-NB spikes influences the measure of similarity, shown in Fig 7D, we reported the cumulative similarity curve from M = 6 to M = 66. The similarity was also computed for pairs of pre-NB activities belonging to the same NB cluster (IN) or among pre-NB activities belonging to distinct NB clusters (ACROSS). Similarity matrices of Fig 7E and 7F were reordered using Ward’s method as criterion for the hierarchical clustering algorithm.

## Supporting information

S1 Appendix The network topology is a determinant of propagating activities. (DOCX)

S2 Appendix Emergence of inhomogeneities in the connectivity of Gaussian graphs. (DOCX)

S3 Appendix Networks statistics in different graph models: a comparison. (DOCX)

S4 Appendix Network burst detection. (DOCX)

S5 Appendix Role of GABA receptor in cell cultures dynamics. (DOCX)

S6 Appendix Role of NMDA receptor in cell cultures dynamics. (DOCX)

S7 Appendix Ignition sites co-localize with functional communities. (DOCX)

S8 Appendix Relations between pre-NB activities and network burst propagations. (DOCX)

S1 Table Average values for graph properties (n = 10 graph realizations). (PDF)

S2 Table Parameters of AdExp for the two neuronal population of the model. (PDF)

S3 Table Parameters of the modeled synapses. (PDF)
