# Supplementary material for: Recurrently connected and localized neuronal communities initiate coordinated spontaneous activity in neuronal networks
Source: PLoS Comput Biol. 2017 Jul 27;13(7):e1005672. doi: 10.1371/journal.pcbi.1005672 (PMC5549760; doi:10.1371/journal.pcbi.1005672)
Supplement: S1 Appendix — (DOCX) [file pcbi.1005672.s001.docx]

# S1 Appendix - The network topology is a determinant of propagating activities

In order to test the role played by the network topology (Fig S1A) on the spontaneous spiking activity (Fig S1B,C) we tested different connectivity rules and their consequences on the propagation of spiking activity during a network burst (Fig S1D). The random topology disregards any spatial constraint (i.e. the connection probability is independent of the mutual distance). In the radius graph, all neurons are connected to each other within a fixed radius (Fig S1, second column). Instead, in the Gauss graph the connection probability is inversely related to the reciprocal distance among the neurons (i.e. the probability decays as a function of distance according to a Gaussian function, Fig S1, third column), see Fig S1A for small representative networks with the same neuronal displacement.

We further considered three different preferential attachment graphs: (i) the canonical PA-node degree model (PAnd, N=4096,m=15 [1], Fig S1 fourth column), in which the preference of attachment is towards the nodes with a high degree, (ii) the PA-position model (Pap, Fig S1 fifth column) in which the preference of attachment is towards the closest neurons (k-nearest neighbours in euclidean space [2], N=4096, k=15), (iii) the PA-node degree & position model (PAndp, Fig S1 sixth column), in which both the degree of the nodes and the position are taken into account (N=4096, m=15, α=σ=1, cfr. [3]).

The random network display emergent NBs that propagate homogeneously across the network (Fig S1, first column). The latter is further confirmed by the center activity trajectory (CAT) of the NB that converges rapidly to the center of the network. Instead, in the radius graph the lack of long-range connections gives rise to activities that slowly propagate across the network. As shown in Fig S1D, the trajectories of NBs elicited by PA models do not resemble the experimental ones. Specifically, in PAnd the CATs converge rapidly to the centre of the network since, similarly to the random graphs, the connectivity does not take into account the spatial arrangement of the neurons (Fig S1, first column). On the other hand, position-based PA networks can generate NBs that propagate across the network. but the corresponding CATs are characterized by many bending points that reflect activities hopping across different regions of the network but this is not observed in experiments.

In addition, the distribution of the firing rate (Fig S1C) in the latter networks displayed spiking activities in well-segregated regions of the network with a large part of the neurons that remained almost silent (dark blue regions) but this is also not observed in experiments (Fig S1C, last column).

Note that, position based PA networks approximate slightly better the experimental firing rate distribution than Gaussian graphs, as quantified by the Kullback-Leibler divergence(Fig S1E). The firing rate distributions (Fig S1B,C) were also in good matching with the experimental data (Fig S1, last column). The match was quantified by the Kullback-Leibler divergence computed respect to the experimental distribution (see Fig S1E) and the other network models.

To summarize, the Gauss graph has several advantages. First, it does not require to impose a specific hub-architecture of the network (as in the PA-models). Second, it represents a good compromise between the extremes: gaussian graphs have a balanced node degree distribution, the presence of many short and few long-range links, recurrent connections and a short path length (slightly worse than PA-position based networks). Third, the simulated CATs resemble the experimental ones.


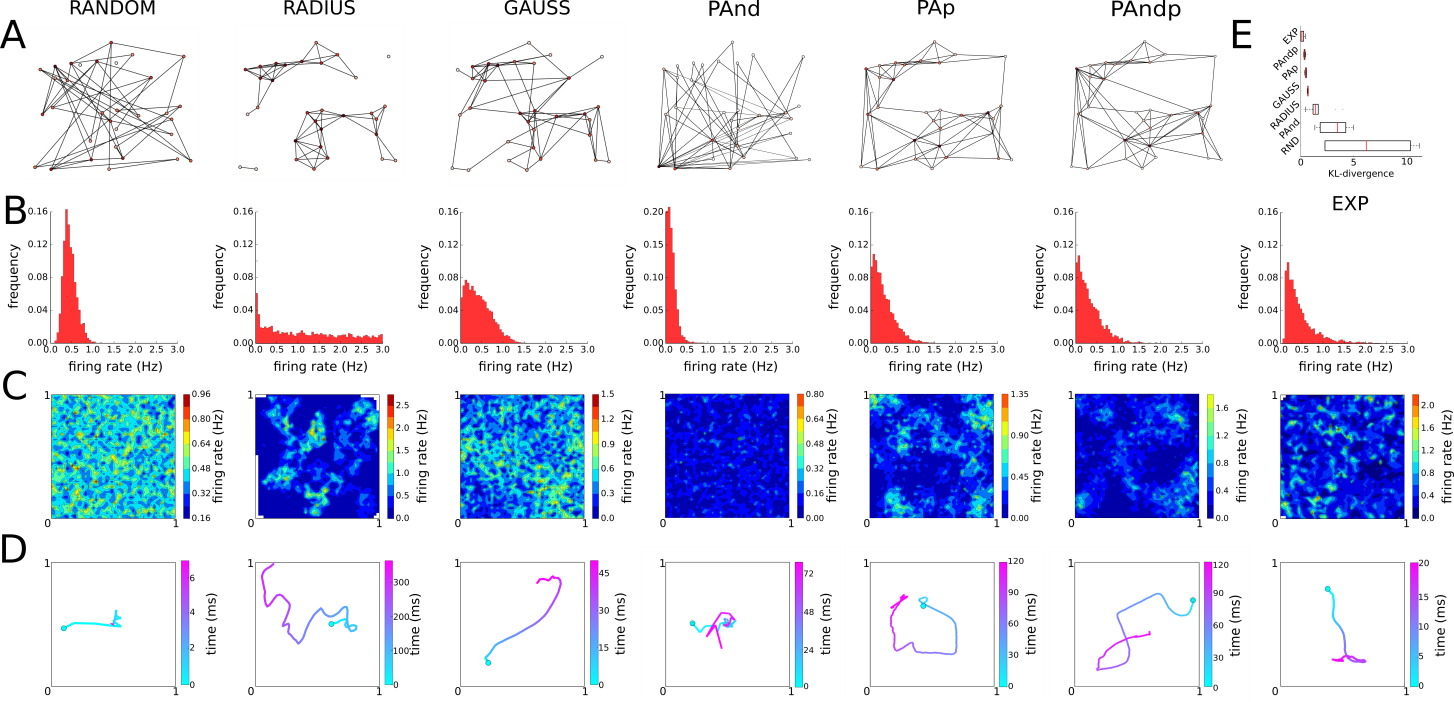


*Figure S1. Network activities for different topologies compared to experimental recordings (EXP): (A) exemplification of the induced topologies by the different connectivity rules for the same set of neurons. (B) firing rate distributions, (C) spatial distribution of the firing rates and (D) representative CATs. In the random graph, the activity spreads homogeneously and quickly across the whole network (first column). In the radius graph, the CAT propagates slowly across the network compared to the experimental data (bottom column). Finally, in the Gauss graph (third column) the CAT compares well with the corresponding experimental ones. The better matching of the CATs is also reflected in the firing rate distributions when compared to experimental ones (E).*

# References

x

| 1. | Albert R, others. Emergence of scaling in random networks. Science. 1999 oct; 286: 509-512. doi: 10.1126/science.286.5439.509. |
| --- | --- |
| 2. | Franklin WR. Computational Geometry: An Introduction (Franco P. Preparata and Michael Ian Shamos). 1988 dec. |
| 3. | Dell'Amico M. Highly clustered networks with preferential attachment to close nodes. In Proceedings of Second European Conference on Complex System; 2006. |

x
