# Supplementary material for: Recurrently connected and localized neuronal communities initiate coordinated spontaneous activity in neuronal networks
Source: PLoS Comput Biol. 2017 Jul 27;13(7):e1005672. doi: 10.1371/journal.pcbi.1005672 (PMC5549760; doi:10.1371/journal.pcbi.1005672)
Supplement: S2 Appendix — (DOCX) [file pcbi.1005672.s002.docx]

# S2 Appendix – Emergence of inhomogeneities in the connectivity of Gaussian graphs

To better visualize how inhomogeneities arise in our model, we applied the Gauss connectivity algorithm to six distinct spatial arrangements of neurons, ranging from a regular equispaced grid (Fig S2A) to a fully random distribution (Fig S2F). In regular grids (Fig S2A-B) the node degree stays constant at the centre of the network and it decreases approaching the border because of a finite size effect (i.e. lack of nearby neurons to connect with). Interestingly, in the random arrangements, clusters of densely interconnected nodes emerged (Fig S2E-F). Importantly, the sparseness in the connections (i.e. low degree of connectivity respect to all possible connections) is a crucial parameter and in the model it was constrained to reflect the node degree found in similar experimental preparations (see Materials and Methods, section Network Topology). To summarize, the much denser interconnected nodes emerge from a combination of factors: 1) the random arrangement of the neurons, 2) the distance dependent connectivity rule, and 3) the sparsity of the connections.

| 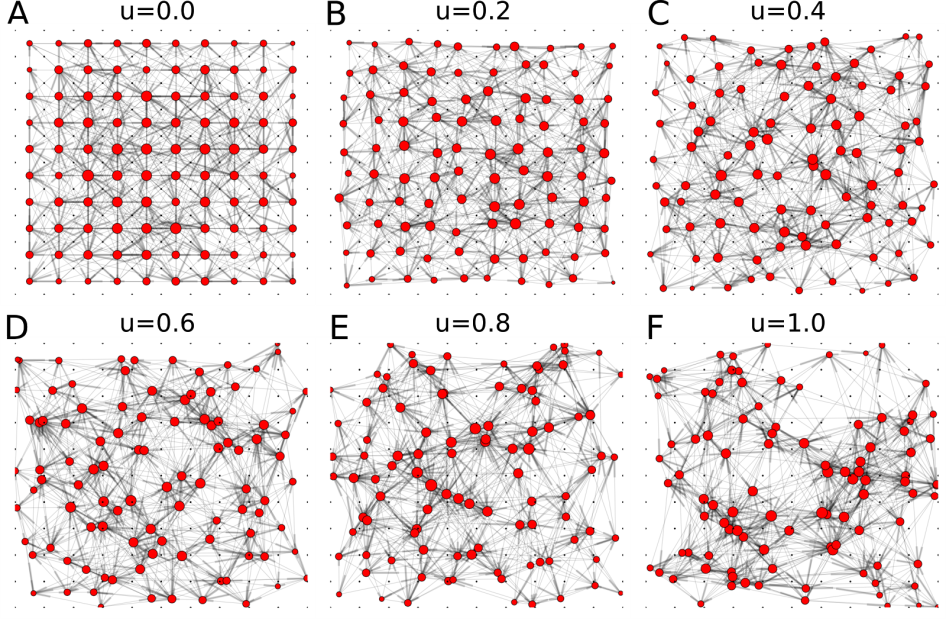 |
| --- |
| *Figure* S2*. Emergence of densely connected microcircuits. Microcircuits with a denser connectivity pattern (the size of each node is proportional to its degree) emerge moving from a regular grid network (A, u=0) to a fully randomized network (F, u=1). All networks consisted of N=100 neurons.* |
