# Supplementary material for: Recurrently connected and localized neuronal communities initiate coordinated spontaneous activity in neuronal networks
Source: PLoS Comput Biol. 2017 Jul 27;13(7):e1005672. doi: 10.1371/journal.pcbi.1005672 (PMC5549760; doi:10.1371/journal.pcbi.1005672)
Supplement: S3 Appendix — (DOCX) [file pcbi.1005672.s003.docx]

# S3 Appendix – Networks statistics in different graph models: a comparison

In Figure S3 we report the statistics of the graph parameters obtained with the connectivity rule implemented in the paper (green lines), the random graph (cyan), the radius graph (pink) and different preferential attachment graphs. The degree distribution of the PA models is characterized by heavy tails (Fig S3A) while the Gaussian and random graphs are characterized by more balanced distributions. In terms of the link length, the random and PA-node degree graphs display a broad distribution (Fig S3B) since they disregard any information on the spatial arrangement of the neurons. The Gauss graph has a lower tail compared to the PA-position and the PA-node degree & position graphs. Since the PA position graphs are inspired by realistic biological connectivity rules one could argue that the Gauss graphs might underestimate the real amount of long connections. Instead, for the radius graph the link distribution is unrealistic by construction. In terms of the clustering coefficient, the PA-position based graphs display the highest values (Fig S3C). Note that, although in the radius graph the nodes are well connected up to a certain radius, the lower clustering coefficient respect to the PA-position graphs can be explained by the lack of long-range connections. Here, the distribution of the clustering coefficient values of the Gauss graph is halfway from the random and the PA-models graphs.

Finally, the distribution of the shortest path lengths of the Gauss graphs is skewed towards the highest values respect to the PA-models, because they have less long-range connections compared to the PA-models (Fig S3D).


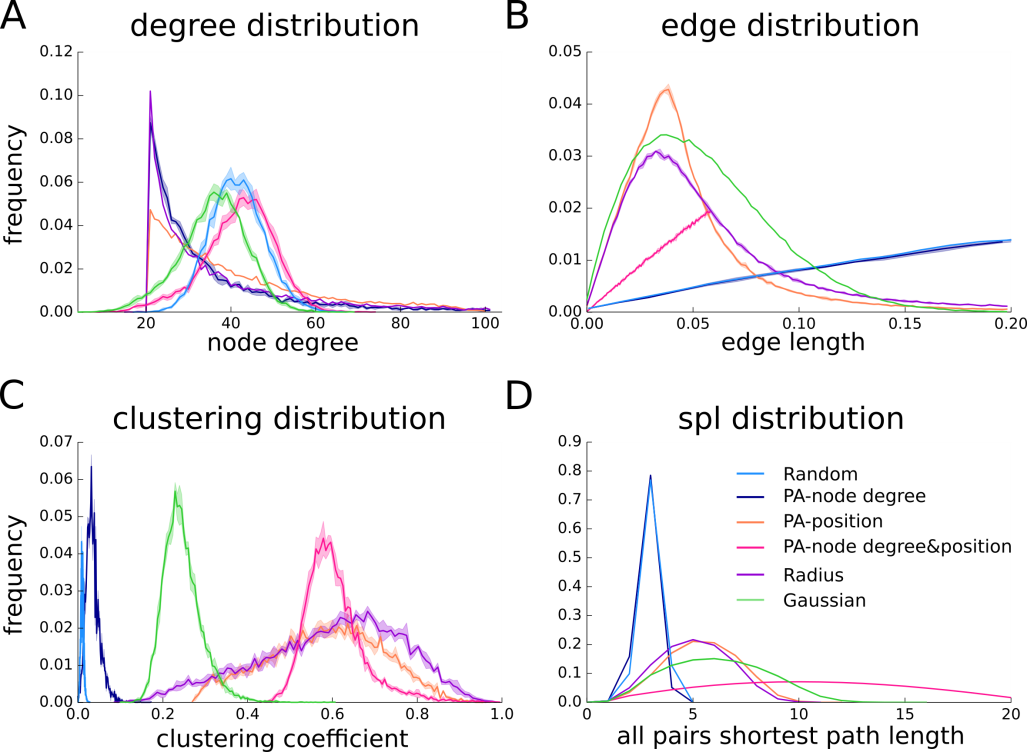


|  |  |
| --- | --- |

*Figure S3. Graph parameters for the different networks. The network parameters of the Gauss graph (green line) are compared to other graph generators in terms of (A) node degree, (B) link length (edge) distribution, (C) clustering coefficient and (D) shortest path (spl) length. Network statistics are presented as mean (solid line) and one standard deviation (shaded area). For each graph generator 5 different networks were built.*
