# Supplementary material for: Recurrently connected and localized neuronal communities initiate coordinated spontaneous activity in neuronal networks
Source: PLoS Comput Biol. 2017 Jul 27;13(7):e1005672. doi: 10.1371/journal.pcbi.1005672 (PMC5549760; doi:10.1371/journal.pcbi.1005672)
Supplement: S4 Appendix — (DOCX) [file pcbi.1005672.s004.docx]

# S4 Appendix - Network burst detection

In the simulated data, the network bursts were detected using a simple hard threshold algorithm (HT algorithm). At first, the network activity is discretized (bin size 10 ms) and for each time bin the number of active neurons is computed. All time bins with more than $5$ active neurons are considered as potential network bursts (NB) event and the timing of each NB is refined by shifting backward the starting point of the NB (up to 50 ms) and forward the end point of the NB (up to 250 ms). The sliding procedure is iteratively applied backward and forward until the count of active neurons is lower than $1\%$ of the total neurons. In addition, in order to detect more reliably the network bursts in experimental datasets, we developed an algorithm (NB-graph) that isolates and tracks the propagating NBs. The NB-graph algorithm takes advantage of the spatial and temporal resolution of the 4096 electrodes APS-MEA by considering the NB as a coherent activity in time and space.

The algorithm works as follows:

1. a set of potential NBs with their reference time ($t_{B}$) are extracted
2. for each putative NB, all spikes falling in the time window ($t_{B}$-50,$t_{B}$+200) ms are analyzed
3. each spike is then considered as a node of a graph and it is connected to another node (i.e. spike) if the temporal and spatial distances are lower than some prefixed thresholds ($\tau_{NB}$=10 ms, $d_{NB}$ =6 electrodes)
4. the NBs are then defined as the largest connected components, with the nodes/spikes falling close in time and space.

Figure S4 shows that the NB-graph algorithm denoises the spiking activity by preserving the propagating nature of the NB. Figure S5 illustrates the performances of the NB-graph algorithm, in different contexts, respect to the aforementioned hard threshold algorithm.

These examples show that the NB-graph algorithm denoises the spiking activity, but it does not alter the propagation of the NB. For instance, when the background activity is low, the NBs are equally well detected by the two algorithms (Fig S5A). However, when the background activity is higher, the NB-graph detects properly the propagation of the NB (Fig S5B,C). A more quantitative analysis performed over a large set of diverse NBs (1606 NBs from 5 different datasets) showed that the number of spikes retained by the NB-graph algorithm correlates well with the one found by the HT algorithm (Fig S5D).


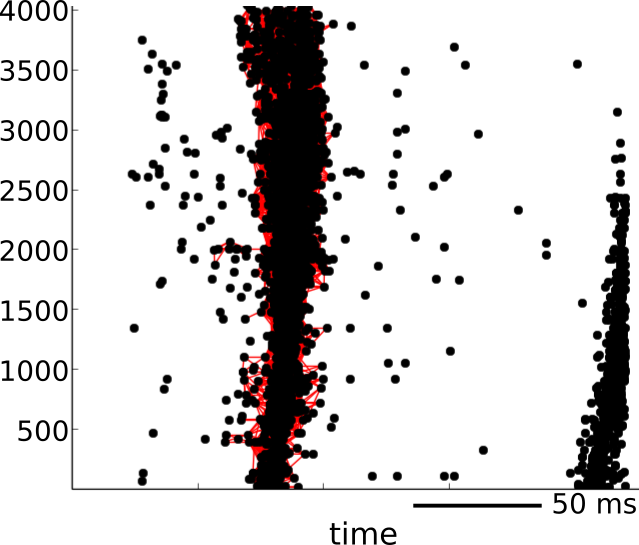


*Figure S4. Detection of a network burst with the NB graph algorithm. The NB graph algorithm aggregates (cfr. red links) the ’nodes’ (i.e. spikes) that are confined in time and space.*


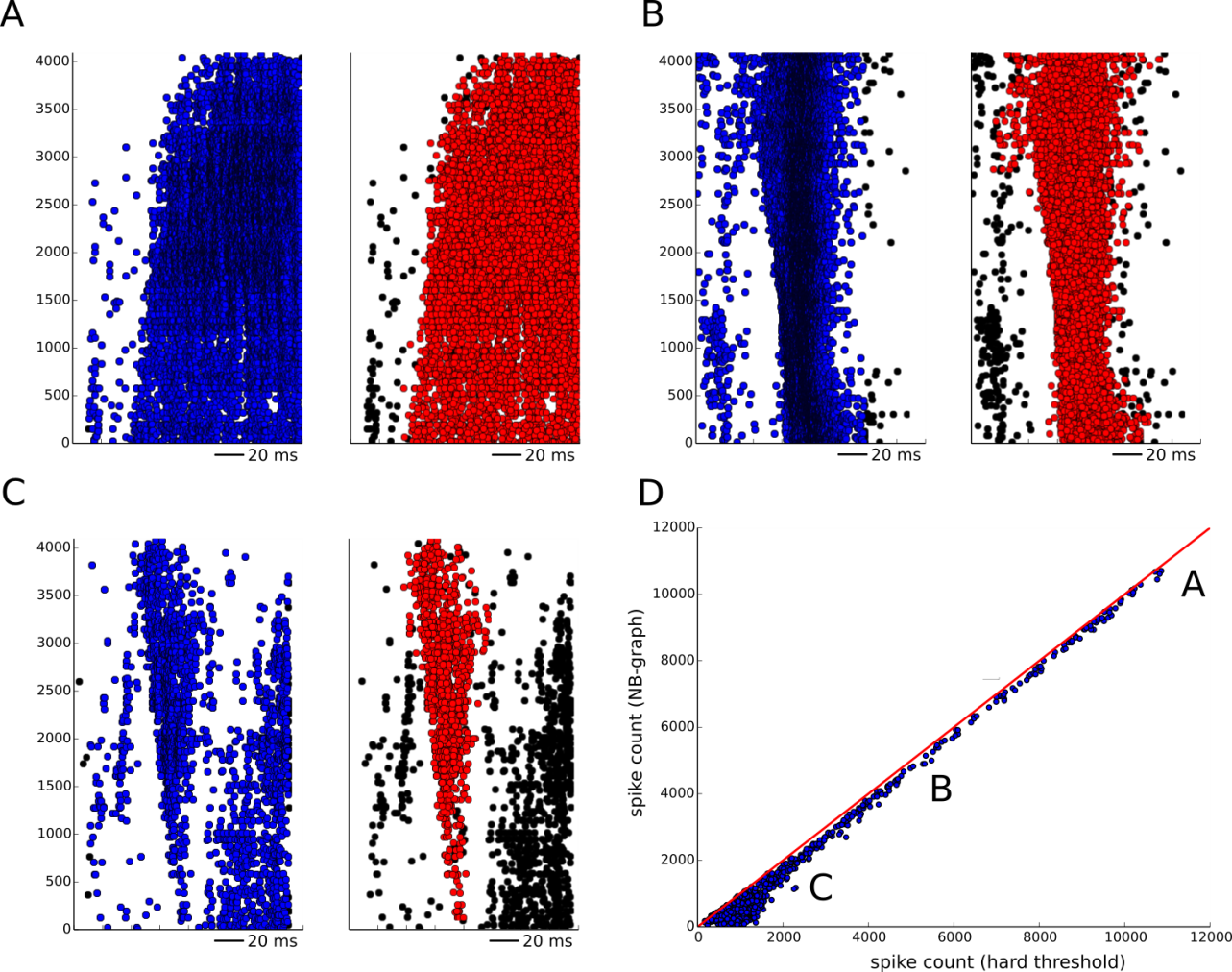


*Figure S5. Comparison of the two network burst detection algorithms. The performances of the NB-graph (red dots) and HT algorithm (blue dots) are compared for different events. As long as the NB event is well defined (A) the two algorithms detect substantially the same spikes. When the background activity is lower (B,C) the NB-graph algorithm detects properly the propagating event (red dots) while it removes the spikes not participating to the NB event (black dots). (D) The NB-graph algorithm filters out the spikes that are not participating to a propagation, but the number of spikes of the NB events still correlate linearly (1606 NBs from 5 different datasets).*
