# Supplementary material for: Recurrently connected and localized neuronal communities initiate coordinated spontaneous activity in neuronal networks
Source: PLoS Comput Biol. 2017 Jul 27;13(7):e1005672. doi: 10.1371/journal.pcbi.1005672 (PMC5549760; doi:10.1371/journal.pcbi.1005672)
Supplement: S5 Appendix — (DOCX) [file pcbi.1005672.s005.docx]

# S5 Appendix - Role of GABA receptor in cell cultures dynamics

Changes in the inhibitory synaptic transmission, as determined by the blockade of the GABA receptors, does not prevent the network to fire bursts [4,5]. The removal of the inhibitory synapses in the model (GABA-OFF, Fig S6), i.e. by setting the GABA conductance to zero, results in faster NBs than what it is observed under control conditions (GABA-ON), as well as in an increased number of spikes that contribute to the NBs (Fig S6A). To explain these changes in the spiking activity, the model suggests that the GABA-OFF condition determines an increased excitation in the network that causes the excitatory synapses to depress strongly with a consequent shortening of the time window on which a neuron is firing. A previous work [6] has shown that a local inhibition of GABA can determine a persistent change in the spatiotemporal structure of NBs. Inspired by this work, we compared the clustered NBs in the GABA-ON and GABA-OFF conditions. In order to cluster the NBs considering the different duration of these events across the tested conditions, we equalized the temporal scales of the NBs. The analysis of the NBs similarity shows that most of the NB propagations are still comparable across the two phases (Fig S6B-C), with a few exceptions (i.e. class marked with an asterisk in Fig S6D).

Therefore, these results show that the removal of the inhibitory circuitry does not disrupt the spontaneous generation of NBs. Inhibition, instead, regulates the duration, the strength, the occurrence of NBs (i.e. the frequency) and the stability of the propagation paths (i.e. lower variance among CATs of the same cluster and less convoluted trajectories). Indeed, the higher intraburst firing rate and the consequent shorter NB duration contributes to better define the trajectory (i.e. higher number of sampling points).

.

#
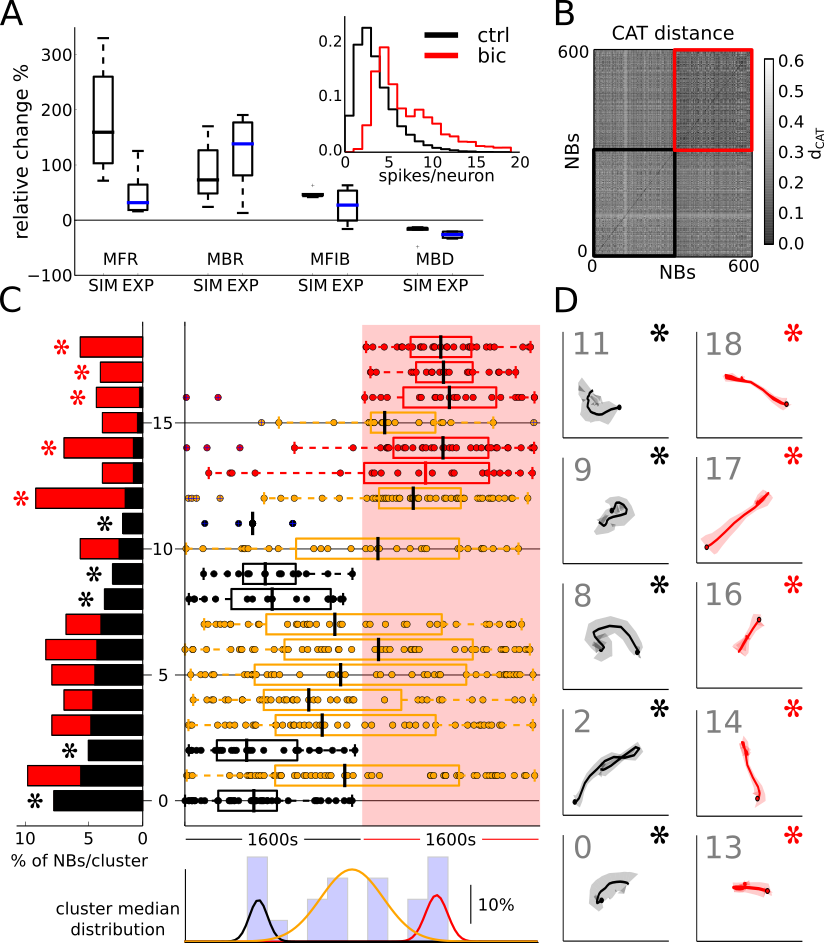


*Figure S6. Role of GABA inhibition on network burst propagations. Inhibition-ON (black, control) and inhibition-OFF (red, blockade of inhibition g_GABA_ = 0). (A) The model predicts the changes in spiking statistics observed in experiments (P-values: 0.052, 0.473, 0.189, 0.449, t-test independent, n=4 experimental recordings, n=5 simulations). When the inhibition is removed, the spike count per neuron during NBs displays a shift toward higher values (inset). (B) Matrix of CATs similarity of the CATs across the GABA-ON (black square) and GABA-OFF condition. (C) Clustering analysis of the CATs in inhibition-ON/OFF phases. Most of the CAT clusters are populated by NBs of the two phases while some clusters are representative of the inhibition-ON phase (e.g. cluster IDs 0, 2) and others of the inhibition-OFF phase (e.g. cluster IDs 16, 17). The Gaussian curves are determined by fitting Gaussian Mixture Models and minimizing the Akaike criterion. (D) The CATs clusters that appear exclusively during the GABA-ON or GABA-OFF phase (mean: thick line; standard deviation: shaded area). Without inhibition, the trajectories are more regular (mostly straight lines) and stereotyped (lower variance).*

# References

x

| 1. | Mack CM, Lin BJ, Turner JD, Johnstone AFM, Burgoon LD, Shafer TJ. Burst and principal components analyses of MEA data for 16 chemicals describe at least three effects classes. Neurotoxicology. 2014 Jan; 40: 75-85. doi: 10.1016/j.neuro.2013.11.008. |
| --- | --- |
| 2. | Suresh J, Radojicic M, Pesce L, Bhansali A, Wang J, Tryba AK, et al. Network Burst Activity in Hippocampal Neuronal Cultures: The Role of Synaptic and Intrinsic Currents. J Neurophysiol. 2016 Mar; 115: 3073-3089. doi: 10.1152/jn.00995.2015. |
| 3. | Baruchi I, Ben-Jacob E. Towards neuro-memory-chip: Imprinting multiple memories in cultured neural networks. Phys Rev E Stat Nonlin Soft Matter Phys. 2007 May; 75: 050901. doi: 10.1103/physreve.75.050901. |

x
