# Supplementary material for: Recurrently connected and localized neuronal communities initiate coordinated spontaneous activity in neuronal networks
Source: PLoS Comput Biol. 2017 Jul 27;13(7):e1005672. doi: 10.1371/journal.pcbi.1005672 (PMC5549760; doi:10.1371/journal.pcbi.1005672)
Supplement: S6 Appendix — (DOCX) [file pcbi.1005672.s006.docx]

# S6 Appendix - Role of NMDA receptor in cell cultures dynamics

In order to better understand how the bursting activity is shaped by NMDA receptors, we characterized the firing regimes with and without NMDA currents (Fig S7A-D). Our simulations show that in the absence of NMDA, the closure of an NB is mainly due to the spike-triggered adaptation (dashed black line Fig S7E), that drives the neurons into a hyperpolarized state (membrane potential, green line, Fig S7E). The NMDA current represents a good candidate to trigger sequences of NBs, called superbursts (Fig S7F-G) for several reasons In fact, first, the time-scale of the NMDA current is compatible with the inter-NB interval of a superburst. Second, the build-up of the NMDA current keeps the neurons in a depolarized state at the end of an NB and balance the hyperpolarization determined by the spike-triggered adaptation (Fig S7G). Third, when the membrane potential of a neuron is below the magnesium block potential, the contribution of the NMDA becomes negligible. It is important to highlight that these three mechanisms act synergically to generate superbursts in the model. The first one is required to match the experimental time-scales, the second one provides an explanation for the onset of consecutive NBs, while the third one is essential to close the superburst. The closure of an NB event (i.e. either single NBs or superbursts) is governed by the same mechanism and it occurs when the adaptation current prevails over the depolarizing one (Fig S7H).

We provide additional arguments that indicate the NMDA as the main determinant of the superburst regime. In [7] the unblock of the NMDA receptor (reducing the magnesium concentration) resulted in an increased NBs rate. Interestingly, other possible drivers of superbursts were suggested by different authors that included the asynchronous release of neurotransmitters, strongly facilitating synapses and a reverberating activity induced by the higher synaptic density at the border of a network. The asynchronous release of neurotransmitters together with the intrinsic recurrent connectivity of neuronal networks is an appealing mechanism [8] since it could provide a steady sub-threshold current that resembles the one provided by NMDA. However, in contrast to our findings, it was also pointed out [9] that the NMDA does not play an important role in sustaining reverberating spiking activities. We interpret this discrepancy respect to our findings as presumably resulting from the younger age and the lower cellular density of the cell cultures used in their experiments. The synaptic facilitation was suggested from modeling results [10]. However, patch clamp experiments on cultured hippocampal neurons have shown that both the release probability and the facilitation time constant at the excitatory synapses is higher [11] than the one hypothesized by the authors. In addition, as observed in experiments [12], as well as in the model (see Fig S6A, inset), neurons fire only a few spikes during a network burst and this prevents a robust build-up of synaptic currents, as it would be required when synaptic facilitation takes place. Finally, another study [13] indicated that the higher synaptic density at the border of the network (i.e. at the border of the plated region) could reverberate the spiking activity and consequently give rise to superbursts. However, our cultures are typically plated beyond the active area (i.e. the electrode array area). Additionally, since our cultures grow without any spatial constraint, the synaptic density likely decreases at the border of the network. This is in clear contrast with a previous work [13], where cell cultures were confined by a physical barrier that gave rise to a higher synaptic density on the border of the network. Although we cannot completely exclude a contribute from the border of the network, in our experiments, the NBs always originated in the observable active area, rather than from the borders of the network. This experimental observation is also in line with our modeling results.

The relevance of the spike-triggered adaptation in controlling the closure of NBs was tested by removing its contribution (b=0, c.f. “spiking neuron model” in Materials and Methods). Under this extreme condition, the network generated never-ending NB activity and the neurons fired indefinitely at a high rate. An additional factor contributing to the closure of NBs is the recovery time constant. In fact, if the excitatory synapses recover too quickly from synaptic depression, the whole network starts to fire at a high frequency. In this situation, the network remains in a hyper-excited state because the synaptic current increases superlinearly with respect to the adaptation current and it cannot be compensated by any other mechanisms. Thus, the computational investigation has shown that the activation and the kinetics of the NMDA current provide the sufficient ingredient to sustain the superburst firing regime.


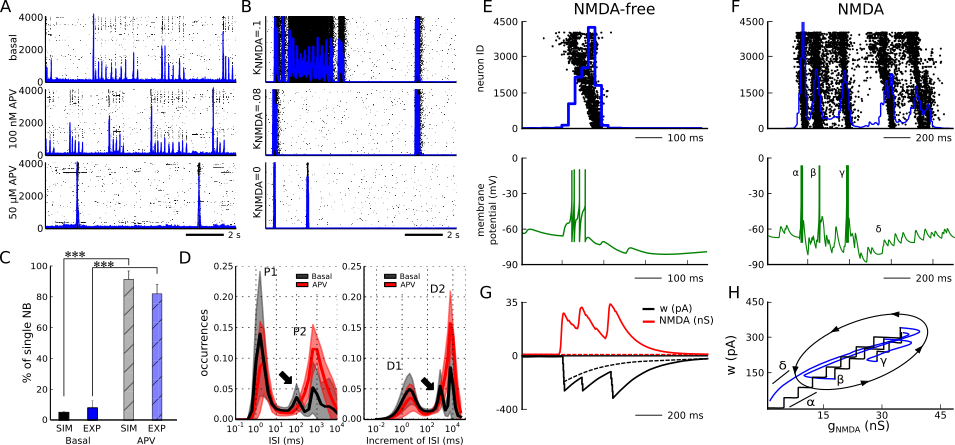


*Figure S7: The NMDA receptor plays a key role in the generation of superbursts.*

*(A) Consecutive NBs are clearly visible in some experiments (blue line is the spike count over 10 ms time windows) and disappear gradually as the concentration of AP5 (an NMDA receptor blocker) provided to the culture increases (bottom panel). (B) By varying the efficacy of NMDA receptors (*$K_{NMDA}$*) the model mimics the different concentrations of AP5 (i.e. the raster plots of the simulations are in good matching with experiments). (C) In a NMDA-free condition (under APV), the network is characterized by single NBs both in experiments (n=3) and AG-networks (n=10). (D) The firing pattern of the superburst regime (black distribution, n=10 simulations) is characterized by three peaks in the ISI and dISI distributions. The blockade of the NMDA (mimicking APV application) causes the disappearance of the peak (solid arrow) at 100ms (left) and 1000 ms (right) representing the firing between consecutive NBs in the superbursts and between superburst, respectively.*

*(E-H) Role of the NMDA receptor in superbursts. (E) NMDA is blocked (*$K_{NMDA}=0$*) and only single NBs can be elicited (n=3 experimental recordings, n=10 simulations). (F) NMDA condutctances are activated with* $K_{NMDA}=.1$ *(as in B). At the cellular level (traces below), the NMDA sustains the depolarization of the neurons and the rhythmic firing. (G) Interplay of the NMDA conductance (red) and the spike triggered adaptation current (black) of a neuron participating to a NB (dashed lines refer to the NMDA-free, panel E, and solid lines to panel F). (H) Phase plane of the current* $w$ *and the NMDA conductance of activity reported in panel B. (*$\alpha$*) During a NB the currents* $w$ *and* $g_{NMDA}$ *increase (black curve) and the hyper-polarization provided by* $w$ *closes the NB (the NMDA enters magnesium block state). Then, AMPA can drive the potential beyond the threshold of the magnesium block by providing a current that drives the neuron in a repetitive bursting regime (*$\beta\gamma$*). Finally, when the NMDA does not counter-balance anymore the adaptation current, the neuron falls in a highly refractory state and recovers back to the resting potential (*$\delta$*).*

# References

x

| 1. | Maeda E, Robinson HP, Kawana A. The mechanisms of generation and propagation of synchronized bursting in developing networks of cortical neurons. J Neurosci. 1995 Oct; 15: 6834-6845. |
| --- | --- |
| 2. | Volman V, Gerkin RC, Lau PM, Ben-Jacob E, Bi GQ. Calcium and synaptic dynamics underlying reverberatory activity in neuronal networks. Phys Biol. 2007 Jun; 4: 91-103. doi: 10.1088/1478-3975/4/2/003. |
| 3. | Lau PM, Bi GQ. Synaptic mechanisms of persistent reverberatory activity in neuronal networks. Proc Natl Acad Sci U S A. 2005 Jul; 102: 10333-10338. doi: 10.1073/pnas.0500717102. |
| 4. | Masquelier T, Deco G. Network bursting dynamics in excitatory cortical neuron cultures results from the combination of different adaptive mechanisms. PLoS One. 2013 Oct; 8: e75824. doi: 10.1371/journal.pone.0075824. |
| 5. | Marconi E, Nieus T, Maccione A, Valente P, Simi A, Messa M, et al. Emergent functional properties of neuronal networks with controlled topology. PLoS One. 2012 Apr; 7: e34648. doi: 10.1371/journal.pone.0034648. |
| 6. | Lonardoni D, Marco SD, Amin H, Maccione A, Berdondini L, Nieus T. High-density MEAs recordings unveil the dynamics of network bursting events in cell cultures. In 2015 37th Annual International Conference of the IEEE Engineering in Medicine and Biology Society (EMBC); 2015 Aug: Institute of Electrical and Electronics Engineers (IEEE).: 3763-3766. doi: 10.1109/EMBC.2015.7319212. |
| 7. | Gritsun TA, le Feber J, Rutten WLC. Growth dynamics explain the development of spatiotemporal burst activity of young cultured neuronal networks in detail. PLoS One. 2012 Sep; 7: e43352. doi: 10.1371/journal.pone.0043352. |

x
