# Supplementary material for: Recurrently connected and localized neuronal communities initiate coordinated spontaneous activity in neuronal networks
Source: PLoS Comput Biol. 2017 Jul 27;13(7):e1005672. doi: 10.1371/journal.pcbi.1005672 (PMC5549760; doi:10.1371/journal.pcbi.1005672)
Supplement: S7 Appendix — (DOCX) [file pcbi.1005672.s007.docx]

# S7 Appendix – Ignition sites co-localize with functional communities

The functional connectivity analysis revealed that the strongest links tended to cluster in few and distinct sub-regions of the network called functional communities (fCOMs). Interestingly, we found that the network bursts originated exclusively from these regions (FigS8).


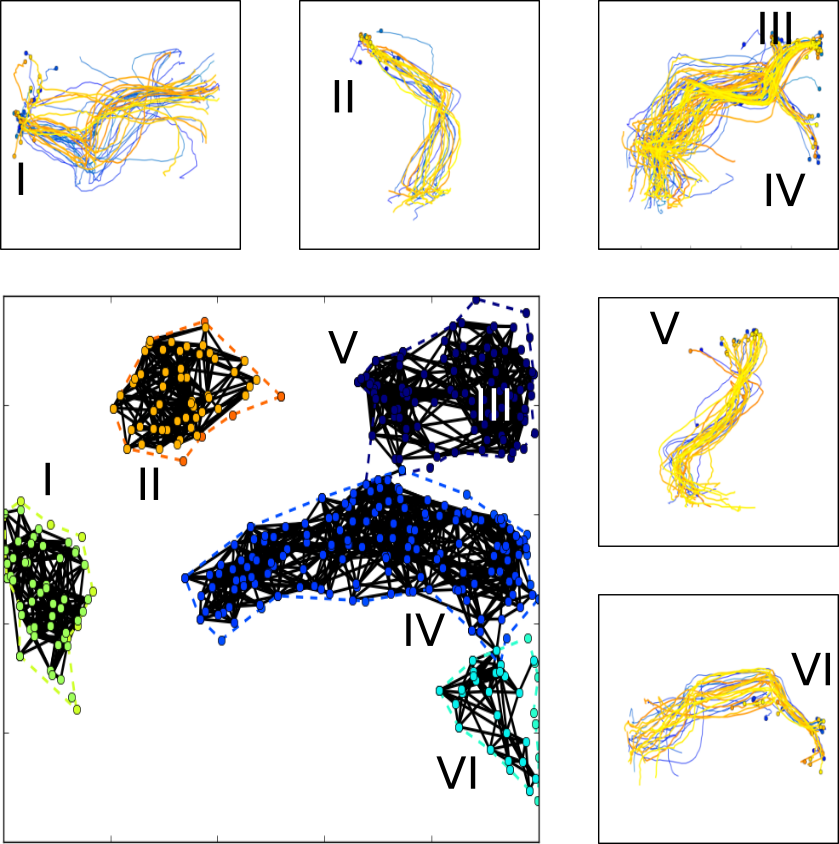


*Figure S8. Network bursts originate from specific regions of the network. The ignition site of the trajectories belonging to the same class of propagations (marked with I-IV) originates from common functional communities (color-coded).*
