# Supplementary material for: Recurrently connected and localized neuronal communities initiate coordinated spontaneous activity in neuronal networks
Source: PLoS Comput Biol. 2017 Jul 27;13(7):e1005672. doi: 10.1371/journal.pcbi.1005672 (PMC5549760; doi:10.1371/journal.pcbi.1005672)
Supplement: S8 Appendix — (DOCX) [file pcbi.1005672.s008.docx]

# S8 Appendix - Relations between pre-NB activities and network burst propagations

The network bursts of a particular cluster (i.e. clustering performed on the CAT) showed that the spiking activity preceding the NBs (i.e. pre-NB spikes) had similar firing patterns for all events of the same cluster. We quantified this observation by identifying common spike motifs in the pre-NB pattern of spikes, named temporal motifs (Fig S9A). This was achieved by running the NB-graph algorithm ($\tau_{NB}$=30 ms and $d_{NB}$=6 electrodes) on the pre-NB spikes of all clustered NBs. Then, for each pair of pre-NBs, the spike motifs were defined as the largest connected sub-graph shared within the pair. This procedure was quite robust and the pre-NB spikes were good predictors of the coming NB event (Fig S9B and Fig 7 of the manuscript). However, it was also found that some pre-NB spikes had either no match with the pre-NB spikes of the other events of the same cluster (FigS9C) while others had multiple matches with different clusters (FigS9D). Thus, the pre-NB spikes cannot be always predictive of the resulting cluster because some events could have been attributed to the wrong cluster (FigS9C) while some spike motifs could instead have been too small (i.e. too few pre-NB spikes).


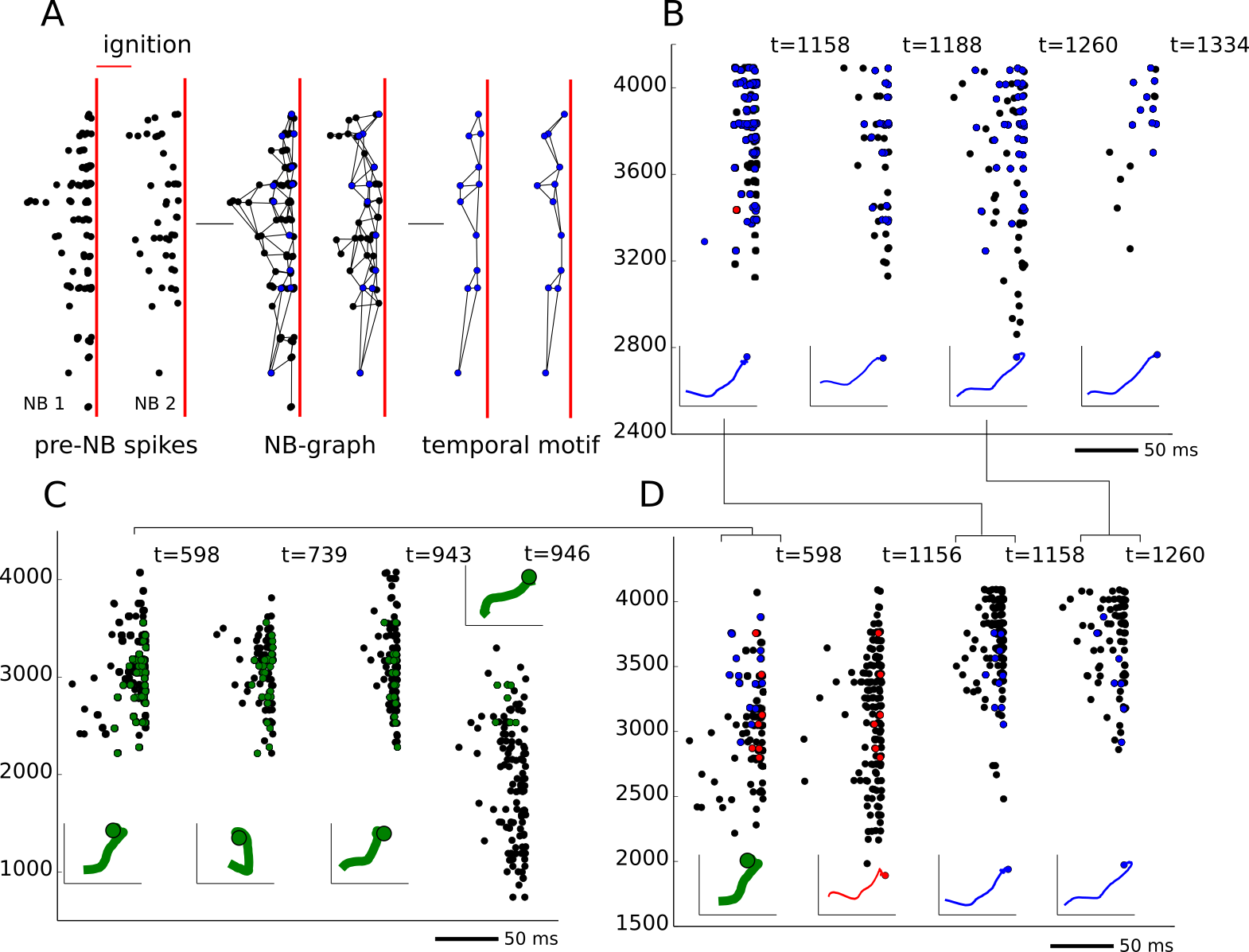


*Figure S9. Characterization of the pre-NB spikes. (A) The pre-NB spikes are processed with the NB-graph algorithm and their temporal motif (see Material and Methods) is reported. (B) Although the pre-NB spikes of four illustrative NBs of the same CAT-cluster may look quite different (different number and structure of spikes) they have a common spike motif. (C) The pre-NB spikes of four NBs of another CAT-cluster. The last NB (t=946 ms) shares few spikes with the other bursts indicating that it was probably assigned to the wrong cluster. (D) The pre-NB spikes may also share some spike patterns with other pre-NBs not belonging to the same cluster. In this illustrative case, some pre-NB spikes of the first cluster (green CAT, the same of panel C) are common to other clusters (denoted with different colors, red/blue CATs and spikes).*
