# Supplementary material for: Recurrently connected and localized neuronal communities initiate coordinated spontaneous activity in neuronal networks
Source: PLoS Comput Biol. 2017 Jul 27;13(7):e1005672. doi: 10.1371/journal.pcbi.1005672 (PMC5549760; doi:10.1371/journal.pcbi.1005672)
Supplement: S1 Table — (PDF) [file pcbi.1005672.s009.pdf]

S1 Table: Average values for graph properties (n=10 graph realizations).

N	σ^2	$degree$	SPL	CC
4096	0.005	41.6 ± 6.4	6.54 ± 0.48	0.24 ± 0.03
