# Supplementary material for: Recurrently connected and localized neuronal communities initiate coordinated spontaneous activity in neuronal networks
Source: PLoS Comput Biol. 2017 Jul 27;13(7):e1005672. doi: 10.1371/journal.pcbi.1005672 (PMC5549760; doi:10.1371/journal.pcbi.1005672)
Supplement: S2 Table — (PDF) [file pcbi.1005672.s010.pdf]

S2 Table: Parameters of AdExp for the two neuronal population of the model.

Neuronal population	a (μS)	b (nA)	C (pF)	g_L (μS)	E_L (mV)	V_T (mV)	Δ_T (mV)	τ_w (ms)	V_{reset} (mV)	I_{bg} (nA)
Inhibitory	2 ± 0.005	0 ± 0	200 ± 0.497	10 ± 0.024	-70 ± 0.173	-50 ± 0.123	2 ± 0.005	30 ± 0.073	-58 ± 0.145	175 ± 0
Excitatory	2 ± 0.005	60 ± 0.150	281 ± 0.711	12 ± 0.025	-70 ± 0.175	-50 ± 0.124	2 ± 0.005	300 ± 0.759	-58 ± 0.147	175 ± 0
