# Supplementary material for: Recurrently connected and localized neuronal communities initiate coordinated spontaneous activity in neuronal networks
Source: PLoS Comput Biol. 2017 Jul 27;13(7):e1005672. doi: 10.1371/journal.pcbi.1005672 (PMC5549760; doi:10.1371/journal.pcbi.1005672)
Supplement: S3 Table — (PDF) [file pcbi.1005672.s011.pdf]

S3 Table: Parameters of the modelled synapses.

Synapse type	τ_{syn} (ms)	E_{rev} (mV)	τ_{rise} (ms)	g_{max} (nS)	τ_{in} (ms)	τ_{rec} (ms)	u
AMPA	3	0	1	48	1	100	0.5
GABA	8	-70	1	48	1	100	0.5
NMDA	280	0	5.5	4.8	1	100	0.5
